# Carbon Nanomaterials-Based Screen-Printed Electrodes for Sensing Applications

**DOI:** 10.3390/bios13040453

**Published:** 2023-04-03

**Authors:** Rafael Matias Silva, Alexsandra Dias da Silva, Jéssica Rocha Camargo, Bruna Santos de Castro, Laís Muniz Meireles, Patrícia Soares Silva, Bruno Campos Janegitz, Tiago Almeida Silva

**Affiliations:** 1 Department of Chemistry, Federal University of Viçosa, Viçosa 36570-900, MG, Brazil; 2 Laboratory of Sensors, Nanomedicine, and Nanostructured Materials, Federal University of São Carlos, Araras 13600-970, SP, Brazil; 3 Federal Center for Technological Education of Minas Gerais, Timóteo 35180-008, MG, Brazil

**Keywords:** disposable electrodes, screen-printing, electroanalysis, carbonaceous nanomaterials, graphene, carbon nanotubes, carbon black, graphitic carbon nitride, carbon quantum dots, biochar

## Abstract

Electrochemical sensors consisting of screen-printed electrodes (SPEs) are recurrent devices in the recent literature for applications in different fields of interest and contribute to the expanding electroanalytical chemistry field. This is due to inherent characteristics that can be better (or only) achieved with the use of SPEs, including miniaturization, cost reduction, lower sample consumption, compatibility with portable equipment, and disposability. SPEs are also quite versatile; they can be manufactured using different formulations of conductive inks and substrates, and are of varied designs. Naturally, the analytical performance of SPEs is directly affected by the quality of the material used for printing and modifying the electrodes. In this sense, the most varied carbon nanomaterials have been explored for the preparation and modification of SPEs, providing devices with an enhanced electrochemical response and greater sensitivity, in addition to functionalized surfaces that can immobilize biological agents for the manufacture of biosensors. Considering the relevance and timeliness of the topic, this review aimed to provide an overview of the current scenario of the use of carbonaceous nanomaterials in the context of making electrochemical SPE sensors, from which different approaches will be presented, exploring materials traditionally investigated in electrochemistry, such as graphene, carbon nanotubes, carbon black, and those more recently investigated for this (carbon quantum dots, graphitic carbon nitride, and biochar). Perspectives on the use and expansion of these devices are also considered.

## 1. Introduction

Electrochemical sensors are devices that have been extensively used in recent years [1]. This fact can be attributed to the low-cost of instrumentation used, the automation, and their performance being equivalent to expensive techniques already consolidated in science [2]. Among electrochemical sensors, the use of screen-printed electrodes (SPEs) has attracted considerable attention because they are versatile, easy to build, reliable, and reproducible. Furthermore, low-cost, robustness, mass producibility, and miniaturization are also examples of the qualities that are achieved when speaking from SPE-type sensors [3].

In recent years, the necessity to detect important chemical species such as biomarkers, pollutants, and drugs, in loco and in real-time, has increased; with that, the need to manufacture electrochemical systems that are portable and easy to obtain has also increased [4]. In this context, the manufacture of SPEs can be performed using a wide variety of conductive components; however, since the discovery of carbonaceous materials, these have been widely explored due to their electrical, optical, thermal, mechanical, chemical, and catalytic properties [5]. Research into the optimal properties of carbon for electrochemical sensors began around the 1960s with the creation of glassy carbon electrodes (GCE) [6]. With the technological advances in morphological characterization, it was possible to discover different forms of carbon, these being useful for the manufacture of devices such as graphene and carbon nanotubes [5,7].

Thus, in the literature, different carbon structures are used for the development of SPEs; in addition to commonly used carbon nanotubes [8] and graphene [9], we also have fullerenes, carbon black, carbon quantum dots, biochar, nanodiamonds, porous carbon, graphitic carbon nitride, carbon nanofibers and activated carbon [10]. It is important to emphasize that it is not only the organization in front of functional the atomic structures but also the interaction with other materials that makes carbonaceous compounds very interesting for the development of these systems. The variety of SPEs not only depends on the materials from which the conductive inks are produced, but also the arrangement of the three electrodes in terms of design and the other materials that can be used to modify the surfaces, depending on the intentionality application [11,12].

A potentially unique aspect of this review is the comprehensive coverage of a wide range of carbonaceous nanomaterials used in the context of the fabrication of screen-printed sensory systems. Well-known materials such as graphene, carbon nanotubes, and carbon black are examined, as are newer materials such as carbon quantum dots, graphitic carbon nitride, and biochar. We also offer perspectives on the use and extension of these devices that could be useful for future research in this field. Overall, this review article provides a thorough and up-to-date overview of the current state of the field, making it a valuable resource for researchers interested in electrochemical SPEs based on carbonaceous materials and their applications.

## 2. Screen-Printing Electrodes (SPEs)

Screen-printed electrodes (SPEs) are devices that have been gaining prominence in recent decades due to their portability, low manufacturing cost, and ease of operation. SPEs are configured as a complete miniaturized electrochemical cell, typically consisting of two or three electrodes, (the working electrode (WE), the counter electrode (CE), and the reference electrode (RE)) [13,14]. Screen printing as a printing method developed mainly after the emergence of screen-printing technology, which allowed for higher quality, device volume, control in printing, and high analytical performance [15]. Screen printing is a popular printing technique used to transfer an image or design onto a surface, such as paper, fabric, or plastic. The technique involves using a stencil, called a screen, to transfer ink onto the surface. For this reason, the name of the technique is screen-printed [16]. However, SPEs can be produced using cutting printers or by hand. The variability in possible designs and sizes makes SPEs flexible and advantageous systems for on-site and real-time detection. The need for only a few milliliters or microliters of sample for detection is another plus point for these systems [17,18]. To manufacture the SPEs, you first have a mesh screen that is the central component where the desired design will be designed. The electrode patterns can be obtained by drawing geometric shapes or by some design software. A substrate, such as paper, plastic, fabric, or tattoo, is placed under the screen, which serves as the base for the SPE. A scraper distributes the ink over the entire print pattern area. After drying and curing at low to high temperatures and adhering the ink to the substrate, the screen is removed, and the SPEs are ready for use [19]. The choice of ink type and rheology are critical factors in developing quality films that spread evenly over the substrate. The quality of the films obtained depends on different factors such as the printer settings, the type of screen, the substrate and its pre-treatment, and the rheology of the ink. To obtain an ideal conductive ink, the rheological properties of the inks, such as viscosity, thixotropy, yield stress, and viscoelasticity, can be evaluated and optimized. In addition, the parameters studied can be correlated with important properties in electrode films, such as electrical conductivity and other electrochemical properties, as well as mechanical strength [20]. As mentioned, the inks used have a conductive character and are made of materials such as gold, platinum, and/or carbon-based materials.

To obtain better electrochemical performance and/or sensitivity, specificity, and stability in the analyses, the modification of the SPEs with different nanostructured materials [21] can be explored. The form and material of the modification depend on the application and the contact medium in which the SPEs will be involved. For example, SPEs can be modified with enzymes, nucleic acids, metals, polymers, and/or electrochemical mediators. Complexing agents, electrochemical signal amplifiers, metals, metal oxides, and metal nanoparticles can be added to the inks used [13,15]. There are four main ways of modifying SPEs. The most widely used is by drop casting (i), in which the modifier material is deposited on the adjacent substrate. It is easy to study the composition of the dispersion in the detection. On the other hand, inkjet printing (ii) allows for a more controllable deposition of the material; in addition, the drops evaporate more quickly. The addition of the modifier material to the ink itself (iii) before the addition to masking the SPEs is another approach, where the viscosity and the other rheological properties of the ink must be taken into account. The final method is the electrodeposition of the modifier material (iv) on the working electrode surface. Modification by electrochemical means allows for greater control of the thickness of the formed film but also presents barriers that hinder its application for large-scale production [15]. The advances in the synthesis of nanomaterials have contributed to the development of SPEs concerning improved electrochemical properties, increased surface area, and higher specificity and sensitivity of the constructed systems [17]. Compared to the method of modifying the SPEs by modifying the conductive ink, modification by adding the nanomaterial to the already screen-printed electrode is a procedure with more steps and requires more reagents. It increases the cost and manufacturing time, and may also present the problem of lack of adhesion of the nanomaterials to the electrode [22].

**Figure 1:**
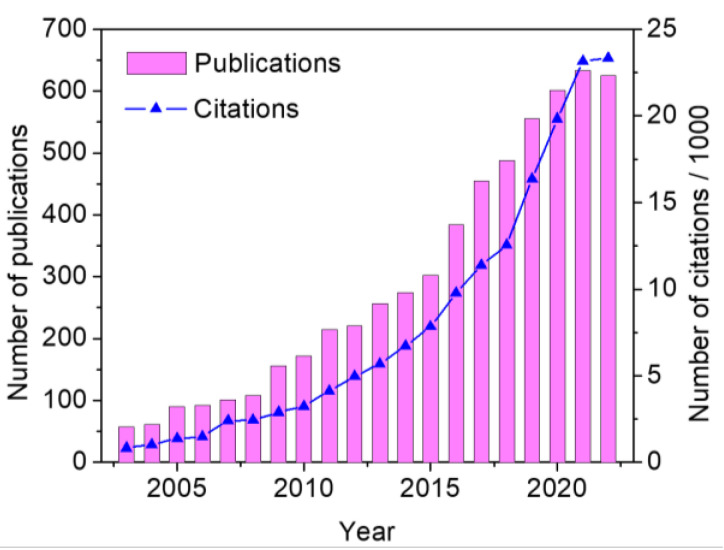
Report of publications and citations provided by the Web of Science database with the keywords “carbon” and “screen-printed electrode”.

As discussed, carbon-based nanomaterials such as graphite, nanotubes, graphene and its oxides, carbon black (CB), and others can be incorporated into the ink of SPEs to improve their physical and electrochemical properties. Figure 1 shows the evolution of the number of publications and citations related to the keywords “carbon” and “screen-printed electrode” over the last few years. As can be seen, this is a scientific field that has been growing sharply, so thousands of works can be found in the last decades. To describe the properties, benefits, and difficulties of modifying SPEs with each of these nanomaterials, the remainder of this review focuses on the evaluation of the application of carbon-based SPEs and their sensing in different areas such as clinical settings, food, and the environment, among others, in the last five years. In addition, novel materials that are gaining prominence for the incorporation and modification of SPEs will be described.

## 3. Carbon Nanomaterials-Based SPEs

Carbon is a nonmetal of great abundance in the biosphere. Its structure can vary from 0D to 3D, giving it great flexibility and versatility in different applications [23]. The application of carbon-based nanomaterials for the construction of electrochemical (bio)sensors allows for a decrease in applied overpotentials, a gain in active surface area, and an improvement in charge transfer between the target species and the transducer. Added to this, the developed devices have the possibility of analyzing multiple species simultaneously, even in a complex matrix. The advantages obtained with the use of these materials come from their physical and electrochemical characteristics, some of which being their sp^2^ C structure (which results in good electrical conductivity and the ability to form good charge transfer complexes), biocompatibility, large surface area, and ease of functionalization with other materials [24].

### 3.1. Graphene-SPEs

Graphene and its derivatives, graphene oxide (GO) and reduced graphene oxide (rGO), have in their structure (2D) sp^2^ carbons with a less or greater degree of oxidation and extension of the π-conjugation system. These materials have been widely applied in the development of (bio)sensors and supercapacitors, given their large surface area, the presence of defects that make it possible to immobilize and anchor nanoparticles and other molecules in their structure, and their high electrical conductivity and mechanical strength. Therefore, one of its applications is as an electrode material of SPEs for the detection and quantification of the most diverse analytes, as summarized in Table 1. The different applications shown in Table 1 are discussed in detail in the next subsections.

#### 3.1.1. Metals

**Figure 2:**
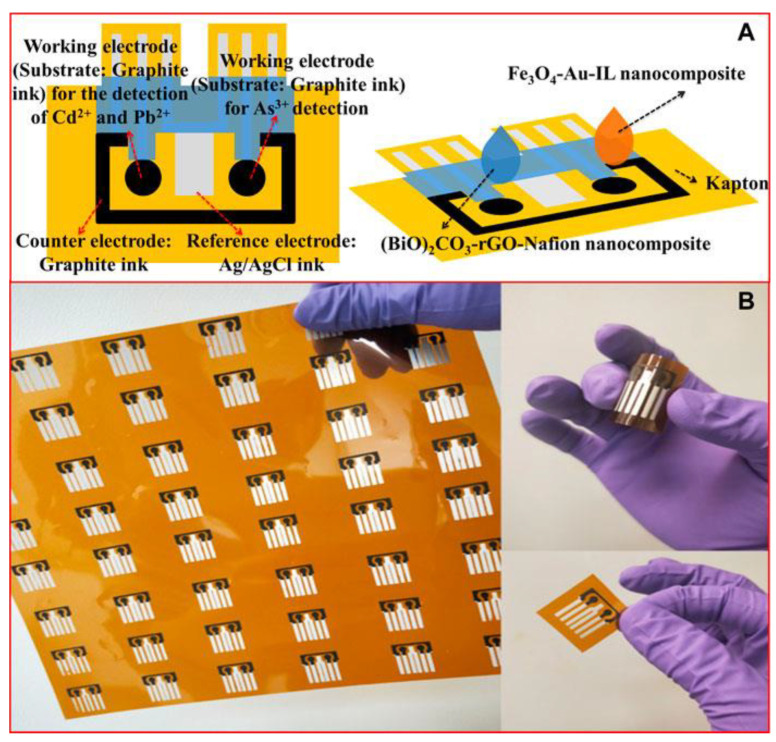
(**A**) Schematic diagram of the SPE. (**B**) Optical images of the SPE. Reprinted with permission from Frontiers from Ref. [27]. Subtitles: IL: L-cysteine.

For the detection and quantification of metals, Mahendran et al. [25] proposed the piperazine-reduced graphene oxide/screen-printed carbon electrode (SPCE), in which graphene oxide was reduced by a green method employing *Hibiscus rosa sinensis* flower extract, and functionalized with piperazine, as an electrode material for Hg(II) detection. The conductive characteristics of reduced graphene oxide added to the presence of piperazine increase the electroactive surface area and present a good interaction with Hg(II), resulting in an electrode with a high sensitivity to the metal studied. Another SPCE system modified by Au/L-cysteine/Fe_3_O_4_/rGO was proposed for the determination of Mn(II) in soil samples [26]. The Fe_3_O_4_ nanoparticles contributed to an increase in the mass transfer of Mn(II) to the sensor, and the L-cysteine electrodeposited on Au stabilized the proposed assembly, contributing to a stable system with a high sensitivity for Mn(II). The sensor showed promise for Mn(II) analysis even in the presence of possible interferents. To perform a multiple compound analysis, Zhao et al. [27] proposed an SPCE with a two-electrode working flow cell, where the sensor nanocomposites were (BiO)_2_CO_3_-rGO-Nafion for Pb(II) and Cd(II) detection and Fe_3_O_4_-Au-IL for As(III) detection. The sensor in SPCE format (Figure 2) with a flow injection system allowed for real-time results and a reduced sample volume, and dispensed a previous treatment of the electrode surface, which represented greater ease and speed of production, in addition to the possibility of using more flexible materials as support for the system.

#### 3.1.2. Agrochemicals

In addition to metals that can directly contaminate the soil and percolate through it to contaminate the groundwater, agrochemicals are also another class of compounds that need to be monitored in the environment. Chromatographic methods present well-established methodologies for the analysis of these compounds; however, they are costly, time-consuming methods that require a lot of time for sample preparation and analysis and a qualified professional to operate the instruments. Due to this, Thanh et al. [28] synthesized a composite material coated with the urease enzyme and deposited it on SPAuEs for glyphosate determination. The incorporation of the carbon materials into the sensor increased the intensity of the response currents of the SPAuEs as it contributed to more catalytic active sites on the sensor surface. The sensor’s high sensitivity to glyphosate, its reproducibility, and its stability make it a viable sensor for use in environmental monitoring. Gevaerd et al. [29] deposited electrochemically reduced graphene oxide onto SPCE to construct a sensor for fenamiphos determination in tomato samples. The sensitivity to fenamiphos oxidation allowed for achieving a low limit of detection and a high-percentage recovery, even in real samples. In another work, atrazine was used as an SPCE modifier material together with reduced graphene oxide for detection at low concentrations of atrazine in complex aqueous samples [30]. The selective cavities in atrazine-rGO/SPCE provided a large surface area for effective atrazine reduction. The authors used pyrrole as a reducing agent for graphene oxide, decreasing ecological impacts and making use of a milder reducing agent. The robustness, sensitivity, and reusability of atrazine-rGO/SPCE are other advantageous features of this system.

#### 3.1.3. Hormones

Hormones are substances that actively participate in different systems of human and animal organisms, and some of them are classified as physiological biomarkers. The low levels of these substances in the body and their participation in different metabolic processes make their monitoring extremely important for health. In this regard, Hao et al. [31] proposed a photoelectrochemical platform based on aptamer and CdTe-GO as a photosensitive SPE modifier material for estrogen detection. The synergy of the photoelectrochemical effects caused the aptamer to show high specificity to the analyzed hormone, being successfully applied to the analysis of food and urine samples. In another work developed by Zhao et al. [32], the SPCE modified with the materials reduced graphene oxide and 5-Amino-2-mercaptobenzimidazol, and gold nanoparticles (AuNPs) were applied for the voltammetric determination of progesterone (P4). The addition of the AuNPs that bound to the 5-Amino-2-mercaptobenzimidazol sulfhydryl group allowed for an increase in the catalytic activity of 5-Amino-2-mercaptobenzimidazol and an increase in the sensitivity of the sensor for P4 determination. The rGO acted as a stable support for the anchoring of the materials in the SPCE, facilitating the assembly of the structure in addition to contributing to an increase in the electrical conductivity of the sensor. Bhansali et al. [33] proposed a modified rGO-AgNPs/SPCE sensor for cortisol determination. In this work, the reduction of GO was performed by atmospheric plasma treatment at room temperature. The hydrogen radicals present in the plasma acted to reduce GO, producing a reduced material with good electrochemical performance and an efficient sensor activity for cortisol detection. The development of this highly sensitive system in a flexible substrate paves the way for further studies investigating a variety of applications.

#### 3.1.4. Dopamine, Ascorbic Acid, Uric Acid and Estriol

Dopamine (DA) plays an important role in the functioning of the hormonal, central nervous, and cardiovascular systems. Ahmad and Kim [34] reported a strategy based on the SPE with tungsten trioxide to determine DA in synthetic urine. The synthesis of the modifier material was simple (sol-gel-method), and the electroanalytical technique applied was square-wave voltammetry (SWV). However, its disadvantage was that DA was not detected in the presence of other analytes, and the interference study was not performed. Within this context, Thirumalai et al. [35] and Zhao et al. [36] made the detection of DA simultaneously with another analyte, an advantage over the work mentioned above. In the approach of Thirumalai et al. [29], the preparation of a new voltammetric sensor was developed by modifying SPCE with a newly synthesized block copolymer poly DMAEMA-b-styrene, as a dispersant for rGO. The sensor was used to determine ascorbic acid and dopamine simultaneously. This sensor exhibited a considerably enhanced anti-interference capability, high reproducibility, storage stability for four weeks, and good peak-to-peak separation for dopamine and ascorbic acid in mixed solutions. A disadvantage was the application of less sensitive voltammetric techniques (cyclic voltammetry, CV, linear sweep voltammetry, LSV). The simultaneous determination of dopamine, uric acid, and estriol were carried out by Zhao et al. [36] using SPE modified with rGO and AgNPs, and differential pulse voltammetry (DPV) as a voltammetric technique. The constructed sensor exhibited good selectivity, reproducibility, stability, and excellent performance in determining dopamine, uric acid, and estriol in synthetic urine samples with excellent recovery. Still, in the context of the determination of more than one analyte, Liu et al. [37] used the SPE made with tyrosinase, chitosan, and rGO for the determination of uric acid or ascorbic acid in human urine samples and showed limits of detection in the nanomolar range. This makes it advantageous when compared to the works cited since in other studies, synthetic urine was analyzed, and the Limit of Detection did not arrive in the order of nanomolar. In addition, the study of interferents was also performed, showing the good selectivity of the sensor used. However, the analysis here was not carried out simultaneously.

#### 3.1.5. Glucose

**Figure 3:**
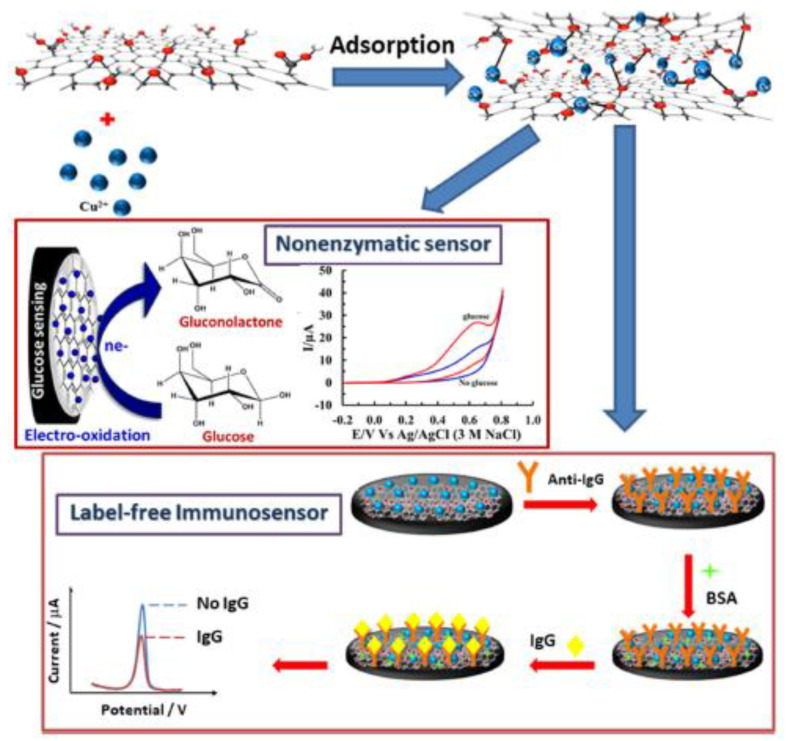
Fabrications of IgG immunosensor and non-enzymatic glucose sensor based on a versatile Cu(II)/GO−modified SPCE. Reprinted with permission from Frontiers from Ref. [44].

Still focusing on graphene-based SPE (bio)sensors, some researchers have reported the determination of glucose [38,39,40,41,42,43,44]. Phetsang et al. [44] presented a new, simple, and versatile electrochemical platform based on SPCE modified with Cu(II)/GO for immuno-sensory detection without an IgG label and nor a glucose enzyme (Figure 3). When compared to the base electrodes, interestingly, the SPCE modified with Cu(II)/GO provides the highest peak of glucose oxidation. The rGO-Au-SPE sensor for the detection of total blood glucose was designed by Ahmadi et al. [39]. The simultaneous use of electrospun nano-fibers and rGO to manufacture ePAD was first reported in this present study, leading to a considerable improvement in analytical performance and structural stability. Modifying SPEs with cobalt oxides and functionalized MoS_2_ and rGO for glucose detection was explored by Li et al. [41]. The modified SPE presented a better-defined reversible CV curve and a higher current response when compared to the unmodified electrode. In addition, a clear amperometric response was shown by the modified SPE, while the addition of interference species did not lead to any noticeable amperometric response, demonstrating the superior selectivity of this sensor. Kailasa et al. [40] demonstrated the practical opportunities for developing electrode materials for environmentally benign, cost-effective, and chemically stable sensors, which can be beneficial for expanding economically viable and enzyme-free electrochemical glucose sensor devices.

#### 3.1.6. Drugs

**Figure 4:**
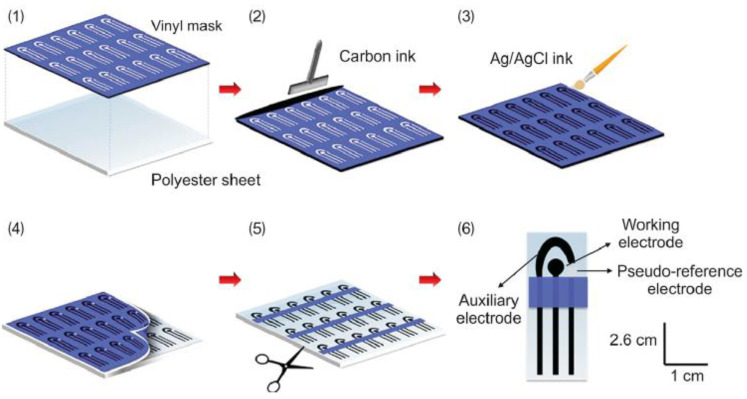
Schematic representation of the preparation of SPE. (1) the vinyl mask was fixed on a polyester sheet (USA Folien Laserjet Clear A4 transparency film); (2) the carbon ink (C2160602D2 from Gwent Electronic Materials Ltd., São Paulo, Brazil) was deposited on the support with a plastic spatula and cured at 90 °C for 30 min; (3) the Ag/AgCl ink (C2051014P10, Gwent Electronic Materials Ltd., São Paulo, Brazil) was applied to the part corresponding to the pseudo-reference electrode, and then the ink was cured at 60 °C for 30 min; (4) removal of the vinyl mask; (5) delimitation of the geometric area of the working electrodes with a rectangular vinyl mask, followed by a heater press and (6) SPE for use. Reprinted with permission from Elsevier from Ref. [48].

Graphene-modified SPEs have been presented as remarkable tools for the electrochemical determination of drugs in different matrices, including foods that may be contaminated with them. The research proposed by Mehmandoust et al. [45] consisted of the development of 2-dimensional graphitic carbon nitride/sodium dodecyl sulfate/graphene nanoplatelets on the SPE for the detection of doxorubicin in biological samples, including human plasma and urine. The great advantage was in the comparison with High-performance liquid chromatography (HPLC), showing agreement in the results. A biosensor to detect the ampicillin presence in buffered and enriched milk samples using a DPV as the voltammetric technique was recently reported [46]. The aptasensor was reusable by simply rinsing with deionized water, remained stable for 15 days during storage, and produced reproducible results. The electrochemical performance of manufactured graphene nanosheets by metal intercalation engineering/SPCE was evaluated by various voltammetric techniques for the pemetrexed anticancer agent as a model target analyte [9]. The detection of pemetrexed anticancer agent in human serum samples was successfully analyzed, and satisfactory recoveries were obtained. Garima et al. [47] developed one SPCE using cobalt oxyhydroxide nanoflakes and rGO to determine the illicit drug clonazepam in different beverages, using DPV as the voltammetric technique. Materón et al. [48] developed a method for the sensitive electrochemical detection of metronidazole in synthetic samples of serum and urine using low-cost SPEs (Figure 4) modified with rGO and C60. A comparison was made between DPV and SWV, and the latter obtained better results and was chosen for the analysis of the respective analyte. It was observed that the intensity of the current for the modified electrode was higher than that of the unmodified electrode. Ali et al. [49] developed a modified SPE with N-Hydroxysuccinimide Crosslinked Graphene Oxide–Gold Nanoflower for the sensitive and selective determination of the antibiotic chloramphenicol. The nanomolar limit of detection and high recovery rates were achieved in different real samples. The better performance of the SPE modified with nanosphere strontium-doped zinc oxide with rGO has proven it to be an efficient electrochemical sensor for the detection of chloramphenicol with the analysis of the actual sample with milk and milk powder samples [50]. Electrochemical sensing analysis was performed with CV and LSV. Compared to an unmodified electrode made with platinum nanoflowers, rGO provided a 5-fold increase in anodic peak current [51]. This newly manufactured electrode not only accelerated electron transfer between the diclofenac and the electrode surface, but also promoted the accumulation of diclofenac on the surface of the electrode, leading to improved electrochemical sensitivity for the quantitative analysis of diclofenac.

#### 3.1.7. Hydrogen Peroxide

Hydrogen peroxide actively participates in various biological reactions in living organisms, and it is used in industry for food manufacturing, chemical engineering, and environmental control. However, prolonged exposure to this chemical can cause cardiovascular disease, skin irritation, and diabetes. To sense hydrogen peroxide present in aqueous samples, Ahmad and Kim [52] explored a composite based on graphite, graphene-like carbon nitride and rGO as an SPE modifier material. The stability and conductive properties of rGO were explored. In this work, the GO was reduced by heat treatment in an autoclave at 180 °C for 8 h and subsequently cooled to room temperature. This methodology dispensed the use of strong reducing agents but still allowed for the construction of a highly sensitive sensor for hydrogen peroxide. Also for the detection of hydrogen peroxide, Zhao [53] proposed an SPE sensor with manganese ferrite nanoparticles (MnFe_2_O_4_) distributed over rGO applying the solvothermal method, which was used as an autoclave for GO reduction and which had a reaction time of 10 h at 200 °C. The SPE with manganese ferrite nanoparticle-decorated graphene nanosheets showed excellent electrocatalytic performance and selectivity towards the studied analyte, even in the presence of potential interfering agents such as glucose, ascorbic acid, and dopamine.

#### 3.1.8. Food Dyes

Food dyes are increasingly employed in the food industry as an alternative to improve the appearance of products to consumers. Because of this, control of the levels of dyes that are added to food is necessary. On this topic, Akkapinyo et al. [54] constructed a rGO-methionine-modified SPCE for the investigation of the dyes amaranth, tartrazine, twilight yellow, and carminic acid. The poly(amino acid) increased the electrocatalytic activity of the sensor and increased the number of active sites for interaction with the target analytes. The rGO, with its large surface area and 2D structure of sp^2^ carbons, acts as a support for anchoring molecules and is a good conductive material. When compared to the classical method of analysis, UV-Vis, the recovery rates from rGO-methionine/SPCE were very close, showing the potential of the sensor as an alternative, cheaper, and simpler method for analysis of the tested dyes. In addition, Wu and Lee [55] developed an SPCE modified not only with rGO, but also with a metal-organic framework based on the nickel (II) and benzene 1,3,5-tricarboxylic acid for the detection of the dyes twilight yellow and tartrazine in beverage samples. The SPE with rGO/nickel(II) and benzene 1,3,5-tricarboxylic acid responded to the analytes at a signal intensity 35.6 and 33.4 times higher for twilight yellow and tartrazine, respectively, compared to the unmodified electrode. The results pointed out that the presence of the rGO composite in the structure improved the conductivity of the system. Another work developed for the simultaneous determination of Sudan I, a food dye, and Bisphenol A, an additive found in food cans, employed CuO/GO nanocomposites in the modification of an SPCE [56]. From the data, it was concluded that the proposed method was simple and promising for the analysis of the species of interest, with good sensitivity and excellent separation between the detection potentials of the analytes.

**Table 1:** Electrochemical sensors based on graphene SPEs.

Analyte	Electrode *	Technique *	Linear Range (µmol L^−1^)	LOD (µmol L^−1^)	Samples	Reference
[Ni(dmgH_2_]	ERGO-AuNPCCAgPPE	SW-AdCSV	---	32.19 μg L^−1^	Drinking water	[57]
4-Cyanophenol	rGO/MFO-2/SPCE	DPV	0.001–700	0.0012	Tap water, industrial river water, and fish	[58]
6-mercaptopurine and 6-thioguanine	RGO-Cu_2_O/Fe_2_O_3_/SPGE	DPV	0.05–400	0.03	Urine and tablets	[59]
8-hydroxy-2′-deoxyguanosine	Ag-TiO_2_-rGO/SPE	DPV	0.05–25	1.0 × 10^−2^	Human urine	[60]
Ampicillin	ErGO-SPE/AuNPs	CV; DPV	1.0 × 10^−5^–1	1.0 × 10^−6^	Buffer and spiked milk	[46]
Arsenic ions	BTA-rGO/SPCE	DPV	2.0 × 10^−3^–4.0 × 10^−2^	2.89 × 10^−3^	HCl solution (0.1 mol L^−1^)	[61]
Atrazine	AIRGOC/SPE	SWV	---	4.0 × 10^−4^	Complex aqueous matrices	[30]
Azathioprine	Mn_2_O_3_–rGO/SPCE	DPV	9–5.73 × 10^5^	4.0 × 10^−3^	Human blood serum and urine	[62]
Beta-amyloid biomarkers	Graphene/rGOSPE/Pyr-NHS	DPV	1.1 × 10^−5^–5.5 × 10^−2^	2.39 × 10^−6^	PBS, Plasma	[63]
Bisphenol A, 8-hydroxy-2′-deoxyguanosine and hydroquinone	ERGO/MWCNTs/SPCEs	EIS; DPV	0.5–25.0, 0.05–50.0 and 0.5–100.0	1.4 × 10^−2^, 3.0 × 10^−3^ and 2.8 × 10^−2^	Human urine	[64]
Brucella	GO/Fe_3_O_4_/MB/Ab2/Ppy	CV; DPV	1.6 × 10^2^–1.6 × 10^8^ CFU mL^−1^	2.2 × 10^2^ CFU mL^−1^	---	[65]
Carbaryl	rGO/AuNP/Nafion	DPV	0.5–250	0.2	River and tap water	[66]
Cd(II) and Pb(II)	Nafion/rGO-MWCNTs-COOH/SPCE	SWASV	8.9 × 10^−4^–2.8 × 10^−2^ and 4.98 × 10^−4^–1.5 × 10^−2^	3.6 × 10^−4^ and 9.7 × 10^−5^	Tap water and lake water	[67]
Cd(II) and Pb(II)	Bi/LC-rGO/DSPE	LSV; DPV	8.9 × 10^−3^–0.27 and 4.8 × 10^−3^–0.14	8.9 × 10^−6^ and 3.9 × 10^−6^	Decorative materials	[68]
Cd(II), Pb(II) and As(III)	(BiO)_2_CO_3_-rGO-Nafion	ASV	0–0.440–0.240–0.67	7.12 × 10^−3^; 5.79 × 10^−6^; 3.20 × 10^−2^	Water	[27]
Chloramphenicol	SPE/rGO–NHS–AuNFs	CV	0.05–100	1.0 × 10^−3^	Blood serum, poultry feed, milk, eggs, honey, and powdered milk	[49]
Chloramphenicol	Sr-ZnO@rGO/SPCE	CV; LSV	0.190–410.84	0.131	Milk and powdered milk	[50]
Clonazepam	CoOOH-rGO/SPCE	DPV	0–350	3.8 × 10^−2^	Beverages	[47]
Cortisol	GO-AgNPs/SPCE	CV	---	---	---	[33]
Dexorobucin	2D-g-C_3_N_4_/SDS/GNPs/SPE	DPV	0.03–13.5	0.01	Human plasma and urine	[45]
Diclofenac	PtNFs/rGO	CV; DPV	0.1–100	4.0 × 10^−2^	Human urine	[51]
DNA	ErGO + AuNUs	DPV	5.0 × 10^−10^–9.5 × 10^−7^	1.6 × 10^−10^	Doxorubicin	[69]
Dopamine	rGO-500/SPCE and of rGO-600/SPCE	CV; DPV	0.5–20 and 0.5–20	1.11 and 1.23	---	[70]
Dopamine	WO_3_/SPE	SWV	---	0.87	Urine	[34]
Dopamine	tyrosinase/chitosan/rGO/SPCE	CV	0.4–8 and 40–500	2.2 × 10^−2^	Urine	[37]
Dopamine and Ascorbic acid	PDbS–rGO/SPCE	LSV; CV	0.1–300 and 10–1100	0.134 and 0.88	Ex vivo brain tissues	[35]
Dopamine, uric acid and estriol	RGO/AgNWs/AgNPs/SPCE	LSV; CV; EIS; DPV	0.6–50; 1–100 and 1–90	0.16, 0.58 and 0.58	Maternal urine	[36]
E-cadherin	SPCE/rGO/PVA/anti-5mC/BSA/DNA-probe-Fe_3_O_4_-CA nanoparticles	CV; EIS	1 × 10^−4^–20 ng mL^−1^	9 × 10^−5^ ng mL^−1^	---	[71]
*Escherichia coli*	SPCE-PANI-AuNPs	CV	8.9 × 10^3^–8.9 × 10^9^ CFU mL^−1^	2.84 × 10^3^ CFU mL^−1^	Milk	[72]
Estradiol	CdTe-GO/SPE	PEC	4.0 × 10^−8^–1.0 × 10^−5^	1.0 × 10^−8^	Royal jelly, milk powder and urine	[31]
Fenamiphos	ERGO-SPE	CV; SWV	0.25–25.0	0.067	Tomato	[29]
Fenitrothion	GO-CMF/SPCE	CV	---	8.0 × 10^−3^	Water	[73]
Ferulic acid	SPE(a)/rGO-AuNPs	CV	1.0 × 10^−2^–1	3.1 × 10^−3^	Orange peels	[74]
Follicle-Stimulating Hormone	rGO/MWCNTs/Thi/AuNP	DPV; CV; EIS	1 mI U mL^−1^–250 mI U mL^−1^	0.05 mI U mL^−1^	Serum	[75]
Food Colorants	rGO-methionine/SPCE	DPV	1–10 and 10–100 for amaranth, 1–10 and 10–85 for tartrazine	5.74 × 10^−2^, 4.8 × 10^−2^, and 3.6 × 10^−2^	Real	[54]
Ganoderma boninense infection	ZnO-NPs/rGO/SPCE	DPV	---	1.75 mg L^−1^	Oil palm leaves	[76]
Glucose	rGO-Au-SPE		3.3 × 10^3^–2.77 × 10^4^	1.0 × 10^2^	Whole blood	[39]
Glucose	Co@MoS_2_/Rgo/SPE	CV; CA	---	3.0 × 10^−2^	---	[41]
Glucose	PANINS@rGO/SPCE	CV; CA	1.0 × 10^3^–1.0 × 10^4^	3.0 × 10^−2^	---	[40]
Glucose	GOx/AuNP/PANI/rGO/NH_2_-MWCNTs	A	1.0 × 10^3^–1.0 × 10^−4^	64	Human blood; serum	[42]
Glucose	rGO-PEDOT:PSS/SPCE	CV	---	86.8	---	[38]
Glucose and cholesterol	ChOx/Pt/rGO/P3ABA/SPCE	CV	2.5 × 10^2^–6.0 × 10^3^ and 2.5 × 10^2^–4.0 × 10^3^	44.3 and 40.5	Human serum	[43]
Glycoside toxins	GO/AuNPs/MPBA	CV; DPV	10–1000	3.4	Food	[77]
Glyphosate	rGO/DWCNTs/Oct-Fe_3_O_4_/Cs/SPAuE	CV; SWV	5.9 × 10^−7^–5.9 × 10^−3^	4.7 × 10^−7^	River water	[28]
GPC3	GPC/GPC3apt/RGO-Hemin/Au NPs/SPE	CV; EIS; DPV	0.001–10.0 µg mL^−1^	2.86 ng mL^−1^	Spiked human plasma	[78]
H_2_O_2_	g-C_3_N_4_/rGO/SPE	CV; LSV	---	0.09	Water	[52]
H_2_O_2_	MnFe_2_O_4_/Rgo/SPCE	CV; EIS	100–4.0 × 10^3^	5.28 × 10^−4^	---	[53]
Hg(II)	TTU-rGO/CSPE	DPV	0.50–0.0	9.97 × 10^−2^	River water	[79]
Hg(II)	P-rGO/SPCE	DPV	2 × 10^−7^–2 × 10^−6^	5.57 × 10^−8^	HCl solution (0.1 mol L^−1^)	[25]
HPV-18	SPE/rGO, MWCNT, Au nanoparticle, L-cysteine	DPV	1.0 × 10^−11^–1.0 × 10^−5^	5.0 × 10^−11^	Extracted DNA from clinical	[80]
HTLV-1	rGO-PPy-(L-Cys)-AuNPs/SPCE	DPV	1.0 × 10^−10^–100	2.0 × 10^−11^	0.1 mol L^−1^ PBS (pH 6.5) containing 100 nmol L^−1^ oligonucleotides based on TAX gene HTLV-1	[81]
Human T-Lymphotropic Virus-1	rGO-PPy-AuNPs/SPCE	DPV	10^−9^–10^−1^	4.0 × 10^−11^	Peripheral Blood Mononuclear Cells (PBMC)	[82]
Hydrazine	ZnFe_2_O_4_/RGO/SPE	DPV	0.03–610.0	0.01	Drinking water; tap water and river water	[83]
Hydroxylamine	NiCo_2_O_4_/RGO/SPE	DPV	0.007–385.0	2.0 × 10^−3^	Water	[84]
IgG and Glucose	CuII-GO/SPCE	SWV	1.0–500 pg mL^−1^	0.20 pg mL^−1^	Serum	[44]
Levofloxacin	Ag/AgVO_3_/N-rGO/SPCE	DPV	0.09–671	7.92 × 10^−6^	Biological and river	[85]
Linagliptin	CuBi_2_O_4_/rGO@MoS_2_/SPCE	DPV	(0.07–0.5) × 10^−3^	5.7 × 10^−5^	Human plasma, urine and tablet	[86]
L-tryptophan	SPE/rGO/AuNPs	CV; DPV	0.5–500	0.39	Human plasma, serum, and saliva	[87]
Lysozyme	SPCE-Amino-rGO/IL/Amino-MSNs	EIS; DPV	1.0 × 10^−8^–2.0 × 10^−1^ and 2.0 × 10^−8^–5.0 × 10^−2^	2.1 × 10^−9^ and 4.2 × 10^−9^	Serum, tears, urine, wine, and egg white	[88]
Metol	CoMn_2_O_4_RGO/SPCE	DPV	0.010–137.67	0.050	Lake water	[89]
Metronidazole	C60-rGO-NF/SPE	SWV	2.5 × 10^−1^–34	2.1 × 10^−1^	Urine and serum	[48]
Microcystin-LR	rGO/Au/Apt/BSA/Mxene/cDNA-MB	CV; SWV	1.0 × 10^−6^–5.0 × 10^−6^	4.0 × 10^−8^	Tap water and surface water	[90]
microRNA	rGO/Au/SPE	CV; DPV	1.0 × 10^−8^–1.0 × 10^−6^	1.0 × 10^−6^	Saliva	[91]
MMP-1	AuNP/PEI/Rgo/SPE	DPV	1–50 ng mL^−1^		Urine, saliva, bovine serum, and cell culture mediums	[92]
Mn(II)	Au/L-cys/Fe_3_O_4_/RGO	SW-CSV	9.1 × 10^−3^–5.5	---	Soil	[26]
Mycobacterium tuberculosis	NH_2_-rGO/TEMPO-nanocellulose/SPE	DPV	1.0 × 10^−4^–1.0 × 10^−7^	3.14 × 10^−8^	M. tuberculosis.	[93]
Na^+^	AgNPs/GO/SPE	CV	0–1.0 × 10^−5^	9.34 × 10^3^	Fish sauce and seasoning powder of instant noodle	[94]
Nitrite	Au/NiO/rGO/SPCE	CV; DPV; CA	1–500	0.2	Water at different locations in Hainan Province	[95]
Nitrite	ERGO/β-CD/CdS/SPCE	CV	0.05–447	2.1 × 10^−2^	Water	[96]
Nitrite	Ag/rGO/β-CD/SPCE	CV	1–2000	0.24	(Spiked) pickles	[97]
Ochratoxin A	SPCEs/GO/cDNA-aptamer/3D-rGO-AuNPs	CV; DPV	2.5 × 10^−8^–2.5 × 10^−3^	1.2 × 10^−8^	Rice and oat	[98]
Pb(II)	rGO/SPCE	DPV	5.0 × 10^−5^–8.67 × 10^−3^	5.0 × 10^−5^	0.1 mol L^−1^ HCl	[99]
Pemetrexed	M-GRNs/SPCE	DPV	0.05–2.2	9.7 × 10^−3^	Human plasma	[9]
Pork	(SPC-RGO)	DPV	0–10.0 µg mL^−1^	1.76 µg mL^−1^	Pork, chicken, and beef	[100]
Progesterone	AuNPs/AMBI/rGO/SPCE	CV; EIS; SWV	9.0 × 10^−4^–27	2.8 × 10^−4^	Calf serum and milk	[32]
Ractopamine	Fe_3_O_4_/GO-MSPE	CV; DPV	0.05–10 and 10–100	1.3 × 10^−2^	Spiked real pork	[101]
ROS	rGO-CeO_2_@Cyt c hydrogel/SPE	CV; DPV	5–30	0.166, 0.338 and 0.229	PBS solutions (pH 7.4)	[102]
Staphylococcal Enterotoxin B	rGOAuNUs/SPCE	DPV; CV; EIS	5.0 × 10^−9^–5.0 × 10^−7^	2.1 × 10^−10^	---	[103]
Sudan I and bisphenol A	CuO/GO/SPGE	DPV	0.3–700.0	0.093	Ketchup sauce, tomato paste chili powder and water	[56]
Sulfadiazine	AuNP-VS_2_-rGO/SPCEs	SWV	1.0 × 10^−2^–3.45 × 10^−1^	4.4 × 10^−4^	Contaminated water	[104]
Sulfite	rGO/PPy NTs-GSPE	LSV; CV; DPV; CA	0.04–565.0	0.01	Water and apple juice	[8]
Sunset yellow and tartrazine	rGO/NiBTC/SPCEs	DPV	0.05–5.0 and 0.075–5.0	0.025 and 0.05	Drinks	[55]
Tartrazine	Pt/CQDs@rGO/SPCE	DPV	0.01–1.57 and 1.57–9.3	7.93 × 10^−3^	Candy, soft drink, jelly powder and water	[105]
Tetracycline	AdTDPV-ERGO-SPEs	DPV	(2.11 ± 0.25) × 10^−8^–(2.09 ± 1.39) × 10^−7^	12	Milk and water	[106]

* The explanations of the abbreviations in Table 1 can be found in the Supplementary Materials.

### 3.2. Carbon Nanotubes-SPEs

Carbon nanotubes (CNTs) have been known since 1991 with their discovery by Iijima [107], and, like graphene, they are among the most widely explored nanomaterials in electrochemistry, especially for the manufacturing of SPEs. A carbon nanotube is seen as a sheet of graphene rolled around a central axis, with its ends open or closed. The physicochemical features of CNTs are dependent on several factors, such as atomic arrangement, diameter and length of the tubes, morphology, and chemical functionalization [108,109]. CNTs can be classified under different chemical and morphological aspects. In this sense, CNTs are usually differentiated according to the number of concentric tubes, with single-walled carbon nanotubes (SWCNTs, a single sheet of graphene rolled up to form a cylinder), double-walled carbon nanotubes (DWCNTs, two concentric nanotubes), and multi-walled carbon nanotubes (MWCNTs, multiple concentric nanotubes) [110]. CNTs for use as an electrode material normally undergo a treatment/functionalization process to remove metallic impurities from the synthesis process and customize their hydrophilicity. The electrochemistry of CNTs is well-reviewed and grounded in the specialized literature [111,112]. A listing of some recently presented works using CNTs to manufacture SPEs can be found in Table 2.

A survey through Table 2 shows that CNT-modified SPEs are used with different types of electroanalytical techniques to perform the analysis of environmental, drug, forensic, biological, and food interests. In some reports, the characteristics of CNTs are combined with those provided by other materials (e.g., metallic nanoparticles, ionic liquids, graphene, and magnetic nanoparticles) for better electrochemical performance, resulting from the defended synergistic effect.

#### 3.2.1. Environmental Analysis

**Figure 5:**
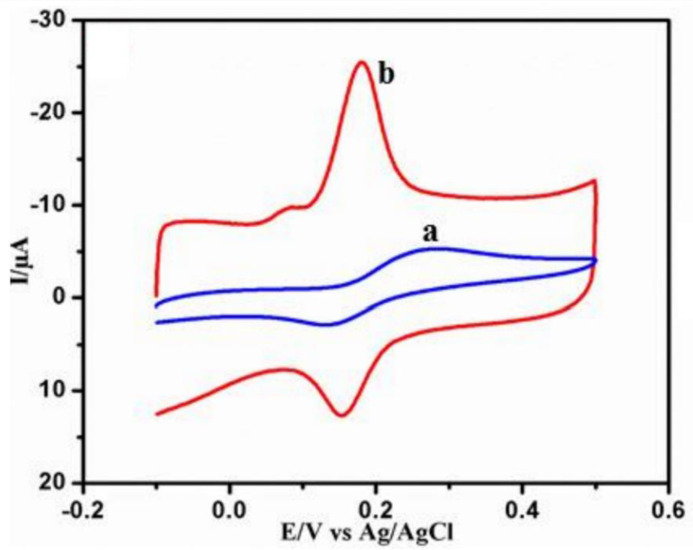
Cyclic voltammetry of bare SPCE (a) and f−MWCNT/SPCE (b) in the presence of 500 μmol L^−1^ CT in 0.05 mol L^−1^ PB solution (pH 7.0) at a scan rate of 50 mV s^−1^. Reprinted with permission from ESG from Ref. [113].

In the context of environmental analysis, phenolic compounds, heavy metals, and pesticides have been determined by CNTs-SPEs sensors in soil and water. Rao et al. [113] reported the determination of catechol, and Figure 5 compares the voltammetric response of catechol on bare and f-MWCNT-modified SPEs; f-MWCNTs refers to nanotubes that have gone through the functionalization process with a mixture of strong acids such as HNO_3_/H_2_SO_4_. From the CVs in Figure 5, the main effects reported by the authors dealing with the modification of SPEs with nanotubes are clear, which is a marked amplification of the peak currents and a diminishing of the peak-to-peak potential separation (Δ*E*_p_), indicating the better reversibility of the analyte’s redox process. The f-MWCNT-modified SPE was applied at the catechol determination by amperometry, with a wide linear range and low limit of detection. Heavy metals such as Cu(II), Zn(II) Pb(II), Cd(II), Tl(I), and U(VI) have already been determined by SPEs modified with CNTs [114,115,116,117]. Chuntib et al. [118] studied the voltammetric response of the herbicide paraquat on SPCEs modified with different nanomaterials, and their superior performance was verified for the modification with a carbon nanotube dispersed in Nafion and ethanol (SPCE-CNT/Nafion). Contributing to the automation of the analyses, interestingly, the authors established a sequential injection-differential pulse voltammetric method to carry out the determination of paraquat.

#### 3.2.2. Human Body Fluids Analysis

**Figure 6:**
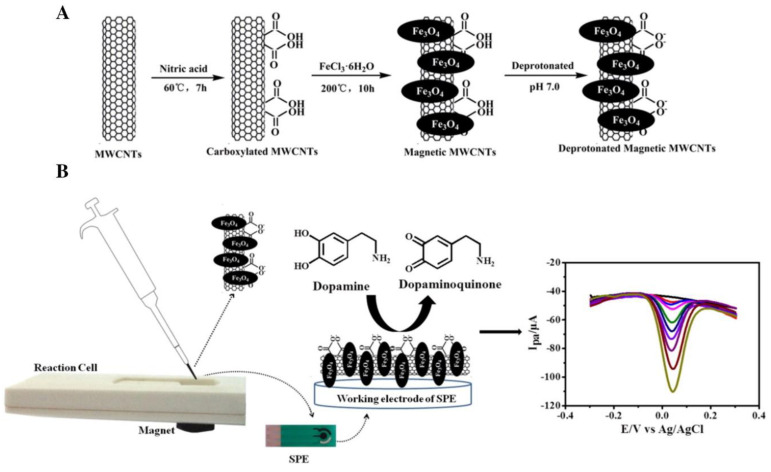
(**A**) Synthetic route of mMWCNTs. (**B**) Magnetism-assisted modification of SPE with mMWCNTs and electrochemical response of dopamine on mMWCNTs/SPE. Reprinted with permission from Elsevier from Ref. [121].

The (bio)sensing of target analytes in human body fluids was carried out by a recent series of research. The voltammetric behavior of the second-generation antihistamine bilastine and its respective voltammetric determination was carried out for the first time by Teixeira and Oliveira [119]. The applied sensor consisted of SPCEs modified by MWCNTs and dihexadecyl hydrogen phosphate dispersion, being dihexadecyl hydrogen phosphate responsible for providing a stable MWCNT dispersion in an aqueous medium as well as homogenous films over the working electrode surface. The MWCNT-SPCE sensor guaranteed a 7-times higher anodic peak current towards antihistamine bilastine irreversible oxidation than non-modified SPCE. Linear sweep voltammetry, as a voltammetric tool at different scan rates, was used to carry out the voltammetric determination of antihistamine bilastine in urine. The authors of this work were concerned with proposing a sensor regeneration procedure, and it was found that a series of washes with deionized water, followed by an air-drying period and electrochemical treatment (CV at 0.1 mol L^−1^ phosphate buffer, pH 6.76, and 10% Methanol), allowed the electrode to be reused at least four times. Thangamuthu et al. [120] presented a study on the determination of bilirubin biomarkers, bringing to light an important aspect—the comparison of the electroanalytical performance of SPEs modified by two different carbon nanostructures: MWCNTs and graphene. Comparatively, the amperometric determination of bilirubin was more effective using graphene-SPE. This type of evaluation is important and often lacking in the literature since the morphologies, structure, and degree of functionalization of carbonaceous nanostructures will affect the electrochemical response of different analytes. Figure 6 presents an approach proposed by Zhang et al. [121] to conduct the voltammetric determination of the neurotransmitter dopamine in human serum by using SPE modified with magnetic MWCNTs (mMWCNTs). The authors present the term “magnetism-assisted modification” to emphasize that the procedure for modifying the SPE with mMWCNTs previously synthesized to present magnetic properties consisted of the simple deposition of a dispersion of mMWCNTs with the consequent fixation of the mMWCNTs on the working electrode by the magnet attached to the back of the SPE. This approach was compared with the classic casting method, and from cyclic voltammetric studies with a redox probe, similar voltammetric responses were obtained for both modified SPEs, which suggests that only the magnetic attraction was enough to maintain the mMWCNTs; this facilitates the modification process without the need to use drying/storage steps. With this novelty highlighted, the sensor turns out to be successfully applied in the quantification of dopamine in human serum samples by square wave voltammetry.

**Figure 7:**
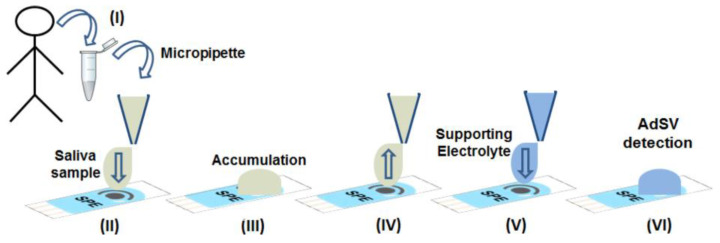
Analysis of authentic saliva samples by the adsorptive stripping wave voltammetry technique using SPE-CNT. Saliva samples were collected (I) using Salivetts^®^, see Section 2.3 (Please, refer to the original work at [122]). (II) the saliva samples (100 μL) with or without addition of modafinil were drop-casted on SPE-CNT using a micropipette; (III) the accumulation step required for AdSV was carried out directly in authentic saliva samples (undiluted) from which the analyte was extracted by adsorption on the electrode; (IV) the saliva samples were removed using a micropipette; (V) the supporting electrolyte was added and; (VI) the detection was performed by AdSV or adsorptive square-wave voltammetry (AdSWV) techniques. Reprinted with permission from Elsevier from Ref. [122].

As previously mentioned, SPE-based sensors are strong candidates that contribute to the detection of analytes of interest in reduced sample volumes and in loco. This is relevant in the case of human body fluids, and the possibility of promoting the detection of analytes in a sample whose collection is less invasive, such as saliva, is of enormous value for the diagnosis of diseases and forensic applications/anti-doping tests. In this regard, a contribution to the field of anti-doping analysis was recently reported by the authors Santos and Compton [122], who used SPEs modified with CNT to perform the determination of the stimulant drug modafinil, which is banned from sports competitions, in saliva samples. This was the first report on the subject, and Figure 7 illustrates the detection strategy followed by the authors. The simplicity of the analytical operation should be noted. Thus, a reduced volume sample of human saliva (100 µL) was first cast on the SPE, followed by the direct pre-accumulation of the analyte (for 20 min), thus without any dilution of the sample. Thereafter, the saliva on the electrode surface is replaced with a supporting electrolyte, and a square wave voltammetry scan was run to perform the anodic stripping of the pre-accumulated analyte molecules. In this way, the adsorptive stripping wave voltammetry technique was applied, which within the strategy followed, allowed for the extraction of the analyte directly from the sample for it to be detected by the voltammetric measurement. Finally, modafinil was determined by the proposed method without interference from electroactive biological analytes such as uric acid and ascorbic acid.

**Figure 8:**
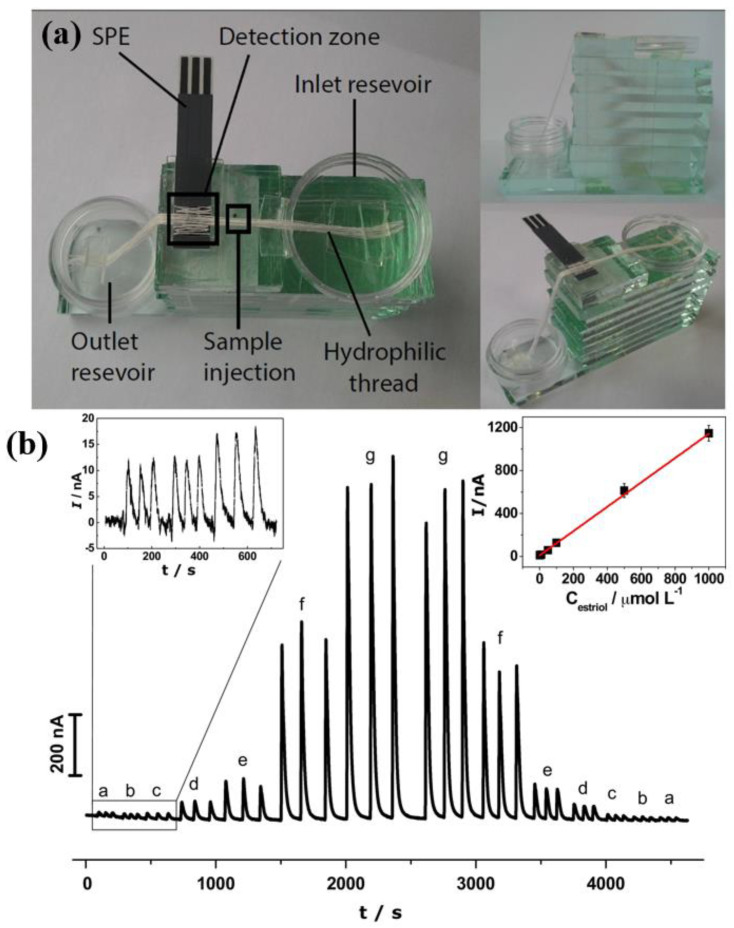
(**a**) Illustration of the thread-based electroanalytical device after the assembly process. (**b**) Successive amperometric response in μTED obtained from injections of 2.0 μL of estriol standard solutions aliquots, varying over a range of (a) 1.0; (b) 5.0; (c) 10.0; (d) 50.0; (e) 100.0; (f) 500.0 and (g) 1000.0 μmol L^−1^, with a flow rate of 0.50 μL s^−1^. Applied potential: 0.75 V. Analytical curve for the amperometric responses in the thread-based electroanalytical device (in detail). Each point is the average value of the six injections for each concentration. Reprinted with permission from Elsevier from Ref. [123].

Earlier in this section, we discussed the analytical strategy of Chuntib et al. [118], who used Nafion and CNT over the SPCE for paraquat determination. It turns out that despite the good analytical performance, this type of methodology requires equipment such as a peristaltic or syringe pump, which can compromise one of the main advantages of the SPEs, which is their low-cost. To get around this issue, very exciting research has been presented with the use of cotton threads as the solution channel for microflow systems applications at a very low cost. One such thread-based electroanalytical device was designed using SPEs modified with CNTs for electrochemical transduction, specifically for the determination of the estriol hormone in pharmaceutical samples [123]. An image of the constructed thread-based electroanalytical device as well as amperograms obtained by the successive injection of estriol standard solutions are both shown in Figure 8. The complete description of the system assembly process can be obtained by the reader in Reference [123]; however, it should be noted that the system was manufactured with simple materials such as glass plates, with non-professionals handling such materials.

#### 3.2.3. Food Products

**Figure 9:**
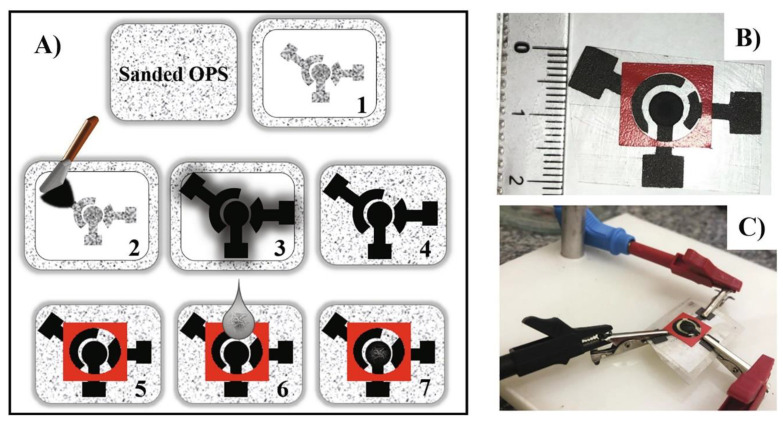
(**A**) Schematic representation of SPE preparation and modification. (**B**) Image of the final device. (**C**) Image of the final SPE connected to the potentiostat. Reprinted with permission from Elsevier from Ref. [124].

Demonstrating the versatility of electrode preparation by screen printing, Araujo et al. proposed an alternative SPE design to the usual one (such as the one shown in Figure 7) for the determination of caffeic acid in food products (teas). Figure 9 displays a schematic of the SPE preparation as well as the final device. In this case, alternative and low-cost materials were used to make the SPEs, including polyester overhead projector sheets as a substrate and conductive ink formulated with graphite powder and colorless nail polish. The SPEs obtained were low-cost and flexible. For better electrochemical performance, once again, MWCNTs were used as a modifier, and the effects of acidic functionalization and size of the MWCNTs on the response of the SPEs were evaluated. The voltammetric method was applied to quantify the caffeic acid in samples of different teas (white, mate, and fennel tea), with excellent recovery percentages.

**Table 2:** Electrochemical sensors based on carbon nanotubes SPEs.

Analyte	Electrode *	Technique *	Linear Range (µmol L^−1^)	LOD (µmol L^−1^)	Samples	Reference
8-hydroxyguanine	MWCNTs-COOH/SPCE	DPV	3.0 × 10^−1^–12	5.7 × 10^−1^	Electrochemical monitoring of stability of 8-hydroxyguanine	[125]
Antihistamine drug bilastine (BIL)	MWCNTs-SPCE	CV and LSV	2.29 × 10^−1^–4.58	1.3 × 10^−1^	Pharmaceutical formulations and urine	[119]
Bilirubin	MWCNTs-SPCE and GO-SPCE	CV	MWCNTs 0.5–500 and graphene 0.1–600	MWCNTs (3.0 ± 0.22) × 10^−4^ and Graphene (1.0 ± 0.18) × 10^−4^	Blood serum	[120]
Catechol	f-MWCNTs/SPCE	CV	8.0 × 10^−2^–725	3.0 × 10^−2^	Water	[113]
Caffeic acid	MWCNT/SPEs	DPV	2.0–50.0	0.2	Tea	[124]
Cd(II)	CuF/GCE and CuF/CN/SPE	ASV	5 × 10^−4^–5 × 10^−1^ for CuF/GCE and 3 × 10^−4^–3 × 10^−1^ for CuF/CN/SPE	CuF/GCE: 1.7 × 10^−4^ and CuF/CN/SPE: 1.3 × 10^−4^	Water	[126]
Cd(II) and Pb(II)	RGO-MWCNT-AuNP/SPE	SWSV	8.90 × 10^−3^–7.12 × 10^−1^ for Cd(II) and 4.83 × 10^−3^–3.86 × 10^−1^ for Pb(II)	6.23 × 10^−3^ for Cd(II) and 1.45 × 10^−3^ for Pb(II)	Soil	[114]
Diclofenac	SPCE/MWCNTs-COOH	DPAdSV	1.0 × 10^−4^–1.0 × 10^−2^	2.8 × 10^−5^	Water	[127]
Dopamine	mMWCNTs/SPE	CV	5–8	4.3 × 10^−1^	Spiked human blood serum	[121]
Dopamine	Modified GCE and SPCE with sodium bis[N-2-oxyphenyl-5-bromosalicylideneiminato-ONO] ruthenate(III), MWCNTs and Nafion	CV, DPV, and flow injection amperometry	up to 326	(7.18 ± 2.61) × 10^−1^	Ampoules of dopamine hydrochloride	[128]
Estriol	CNT-SPCE	Amperommetry; CV	1–1.0 × 10^3^	5.3 × 10^−1^	Commercial pharmaceutical formulation	[123]
H_2_O_2_	SPCE/PAKB NPs/CNTs	CV and amperometry	20–6.48 × 10^3^	2.7	Milk and water	[129]
Imidacloprid	IL-SWCNT/SPC	LSV; CV	11–5.75	8.2 × 10^−1^	Spiked commercial honey	[130]
Indole	MWCNTs-CS/SPCE	CV; DPV	4.27 × 10^−2^–8.54 × 10^−1^	4.27 × 10^−3^	Plasma	[131]
K_4_FeCN_6_, H_2_O_2_ and nicotinamide adenine dinucleotide (NAD^+^/NADH)	MWCNT/GP/SPCE	CV	10–1.0 × 10^3^	3.1 for K_4_FeCN_6_, 7.1 × 10^6^ for H_2_O_2_ and 3.6 for NADH	---	[132]
Kojic acid	MWCNTs-CS/SPCE	DPV	20–5.0 × 10^3^	16	Apple vinegar and Rice vinegar	[133]
Levothyroxine (LT4)	SPCEs, containing CNTs, graphene, and AuNPs individually	DPV	5.0 × 10^−4^–3.0 × 10^−3^ (for CNT)	1.5 × 10^−1^	Diluted fetal bovine serum	[134]
Methyldopa	AuNPs/CNT/SPCE	Flow injection amperometry	2.0 × 10^−1^–100	1.0 × 10^−1^	Pharmaceutical and urine	[135]
Ochratoxin	SPE on PET and PDMS, coated with SWCNTs and immobilized with anti-OTA antibodies.	CV	2.48 × 10^−5^–2.48 × 10^−3^	PET 1.98 × 10^−4^ and PDMS 3.22 × 10^−4^	Grape juice and wine	[136]
Paracetamol, Ibuprofen and Caffeine	SPCE, SPCNTE, SPCNFE, and SPGPHE	DPV	1.32 × 10^−2^–6.62 × 10^−1^ for PA, 9.70 × 10^−3^–4.85 × 10^−1^ for IB and 1.03 × 10^−2^–5.15 × 10^−1^ for CF	5.95 × 10^−1^ for PA, 10.7 for IB, and 1.03 for CF	Spikes tap water and hospital wastewater	[137]
Paraquat	SPCE-CNT/Nafion	CV; DPV	5.4 × 10^−1^–4.3	1.7 × 10^−1^	Natural water	[118]
Piperazine	CNTs-Nafion/GCE and SPCE	LSV; DPV	4.0 × 10^−1^–12	1.1 × 10^−1^	Human Plasma	[138]
Polyphenols content	GCE and SWCNTs-SPCE	SWV	---	---	Red wines	[139]
Secretion of electroactive metabolite(s) in the extracellular matrix	MWCNTs/SPE	CV	1.0 × 10^5^–1.1 × 10^6^ OD600	1.0 × 10^5^ OD600	Bacterial cell suspensions	[140]
Stimulant modafinil	SPE-CNT	AdSWV	7.5–300	2	Saliva	[122]
Tl(I)	SPCE/MWCNTs/BiF	ASV	1.0 × 10^−2^–1	2.8 × 10^−3^	Spiked water from the Vistula River	[115]
Thrombin	SWCNTs/SPCE	CV	1.0 × 10^−1^–1	2.0 × 10^−5^	---	[141]
U(VI)	MWCNTs/SPE (electrode III) and IL-MWCNTs/SPE (electrode VII)	Potentiometry	10–1.0 × 10^5^ for electrode III and 4.7 × 10^−1^–1.0 × 10^5^ for electrode VII	10 for electrode III and 4.7 × 10^−1^ for electrode VII	Water	[116]
Zn(II), Pb(II) and Cu(II)	SPCE/CNTs/AuNP	DPASV	Zn^2+^: 1.51 × 10^−2^–1.84; Pb^2+^: 4.78 × 10^−2^–5.79 × 10^−1^, and Cu^2+^: 1.56 × 10^−2^–1.89 × 10^−1^	Zn^2+^: 1.53 × 10^−2^–5.35 × 10^−2^; Pb^2+^: 7.24 × 10^−3^–2.41 × 10^−2^; Cu^2+^: 1.57 × 10^−3^–5.19 × 10^−3^ for Cu^2+^	---	[117]

* The explanations of the abbreviations in Table 2 can be found in the Supplementary Materials.

### 3.3. Carbon Black SPEs

Carbon black (CB) is a carbonaceous nanomaterial made primarily from carbon, with a minimum amount of oxygen, hydrogen, and nitrogen [142]. It is a by-product formed by the partial combustion of aromatic hydrocarbons in oil furnaces [143]. Lots of nanomaterials, such as carbon nanotubes, graphene, and gold nanoparticles, among others, have been used to modify SPEs’ surface to improve their electrochemical features, e.g., the enhancement of electroactive surface area and electron transfer rate [143] and the reduction of fouling problems [144], as already pointed out previously in this review. Among these nanomaterials, carbon black is particularly advantageous for SPE modification/preparation due to its lower cost (~1 € Kg^−1^), large electroactive surface area, high electrical conductivity, fast charge transfer rates at the electrode surface, and mainly for providing the same or even higher levels of signal amplification of other typically used carbon nanomaterials [145]. Besides that, other benefits of CB as a modifier rely on its ability to form stable dispersions and usability without any further additional treatment [143]. Thus, the cost-effectiveness of CB compared to carbon nanotubes and graphene makes the material an interesting one in the race for the development of efficient and sensitive portable (bio)sensors [142,146]. In this way, CB is a nanomaterial that has been introduced as a good SPE electrode modifier (Table 3).

#### 3.3.1. Drugs

**Figure 10:**
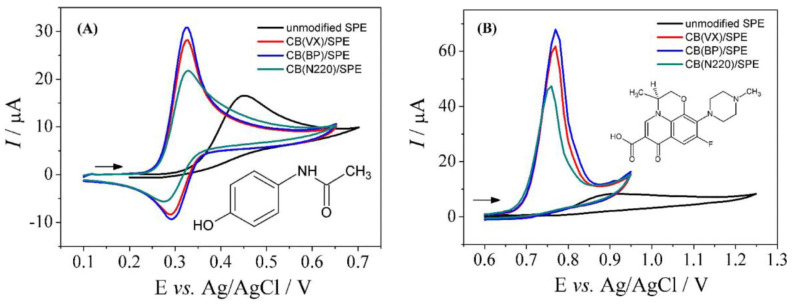
Cyclic voltammograms obtained from the application of the three proposed electrodes: unmodified SPE (black line); CB(VULCAN XC72R)/SPE (red line); CB(BLACK PEARLS 4750)/SPE (blue line) or CB(N220)/SPE (green line) for (**A**) 5.0 × 10^−4^ mol L^−1^ acetaminophen and (**B**) 1.0 × 10^−4^ mol L^−1^ Levofloxacin, in 0.2 mol L^−1^ phosphate buffer solution (pH 3.0), at scan rate (*v*) = 50 mV s^−1^. Reprinted with permission from Wiley from Ref. [147].

There are different types of CBs, with different chemical and physical properties. The usefulness of various types of CBs for electrochemical sensing purposes has recently been compared. One of these studies was developed by Deroco et al. [147], who explored the effect of three different CBs (VULCAN XC72R, BLACK PEARLS 4750, and CB N220) on the simultaneous determination of levofloxacin and acetaminophen by using modified-SPEs. The determination of these drugs is very important since patients under clinical treatment can be treated simultaneously with them; the previous assignment reporting the use of electroanalytical techniques for the simultaneous determination of acetaminophen and levofloxacin explored a glassy carbon electrode (GCE), which is more expensive than SPE and also requires a larger reagent volume. In this context, the electrochemical activity of the three CB types was practically the same (as can be seen from the CVs in Figure 10); even in the SPE, all were successfully applied for the simultaneous determination of acetaminophen and levofloxacin by the square wave voltammetry method.

#### 3.3.2. Phenolic Compounds

It is known that carbonaceous materials can have their properties amplified with the addition of metals, or even the mixture of two or more carbonaceous materials, showing unique and sometimes unexpected detection and catalytic activity properties [148]. In this context, the work developed by Rojas et al. [148] used a CB and a molybdenum disulfide (MoS_2_) hybrid material that was added by drop casting onto the surface of the SPE. In this study, it was shown that CB and MoS_2_, when used individually, had lower analytical performance than that obtained by the hybrid material, proving the excellent affinity of the materials for application in the detection of target o-diphenols in the analysis of extra virgin olive oil and related samples.

#### 3.3.3. Uric acid, Dopamine, Epinephrine, Paracetamol

**Figure 11:**
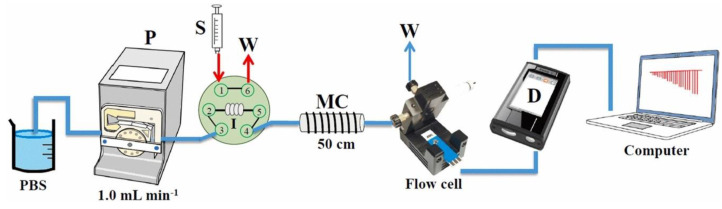
Illustration of FI-Amp system for uric acid determination using a CB-GO modified SPCE (PBS = 10 mM phosphate buffer pH 6.0, P = peristaltic pump, S = standard/sample, I = injection valve (150 μL), MC = mixing coil, W = waste, D = AC impedance electrochemical analyzer-simulator. Reprinted with permission from Elsevier from Ref. [149].

Reanpang et al. [149] developed a simple, rapid, and cost-effective SPE modified with CB and GO for the sensitive determination of uric acid by flow injection amperometric (FI-Amp). Figure 11 displays an illustration of the FI-amp system used to carry out the UA determination. CB combined with GO offers better dispersibility, in addition to a higher specific surface area and electron transfer rate, like all carbons that are applied for this purpose. In addition, CB exhibits its advantages of spherical shape, excellent conductivity, and relatively low price compared to other carbons. In this way, using CB and electrochemical rGO composite, Ibáñez-Redín et al. [150] modified a flexible SPE system made on PET sheets for the detection of clinical and pharmaceutical compounds such as dopamine, epinephrine, and paracetamol, individually and simultaneously. Dopamine and epinephrine are important neurotransmitters present in mammals. These compounds play important roles in the central nervous and endocrine systems. Due to their vital action concerning the heart and blood pressure, for example, dopamine and epinephrine are used in combination for the treatment of hypertension, bronchial asthma, and myocardial infarction. Because they have similar structures and coexist in biological samples, their electrochemical analysis can be difficult due to overlapping peaks. In this context, Fatma et al. [151] developed a system using GO/CB composite based double-imprinted One MoNomer and created a structure like a “cage” over the surface of the SPCE to analyze dopamine and epinephrine simultaneously in aqueous and real samples. This cage, made from CB and GO, makes the SPE system ultra-sensitive and viable in a clinical setting for the simultaneous evaluation of dopamine and epinephrine.

#### 3.3.4. Na^+^ Ion

Some studies have been performed that applied SPE modified with CB as a biosensors. In this sense, Mazzaracchio et al. [144] developed one SPE modified by drop-casting with CB and a selective cocktail membrane applied for Na^+^ ion monitoring in sweat samples. This work aimed to build a wearable sensor that takes advantage of the flexibility and miniaturization of the SPE, and it was reported as the first CB-modified SPE for potentiometric measurements, selecting Na^+^ as the target analyte. As a result, the modified sensor demonstrated long-term potential stability, good shelf life, and resistance to interference from oxygen and light.

#### 3.3.5. Marine Toxins

Another biosensor was explored by Nelis et al. [143], who modified one SPE with CB for the electrochemical detection of the marine toxins okadaic acid and domoic acid, both produced by harmful algal and accumulate in filter feeders and can cause gastrointestinal illness and neurological damage. The CB-SPE was also bio-functionalized with domoic acid or okadaic acid protein conjugates. The innovation of this work was the use of an automated drop-cast of a stable CB dispersion and a good strategy for large-scale sensor production, and detailed stability studies to determine storage possibilities were carried out. Both immunosensors showed excellent storage stability for at least six months, which indicated a possibility for the commercial application of the technology.

#### 3.3.6. Aflatoxin B1

Jafari et al. [145] also developed immunosensors based on CB-SPE. In this work, the purpose was to detect the presence of aflatoxin B1 in cereals. Aflatoxin B1 is a secondary metabolite produced by some fungi and can cause liver cancer in humans. The noteworthy difference of this work from others was the use of a user-friendly smartphone-based magneto-immunosensor that allows for the point-of-need detection of Aflatoxin B1, and also an android application, AflaEsense, designed to display the result in a traffic-light-type format, which is easy to be interpreted by non-expert users. This android application is combined with a commercial miniaturized potentiostat connected to the smartphone. The CB-SPE was also bio-functionalized with Aflatoxin B1 antibody and secondary antibody. As a result, they obtained a highly sensitive and reproducible magneto-immunosensor with false-positive and false-negative rates of less than 5%.

#### 3.3.7. SARS-CoV-2 Coronavirus

**Figure 12:**
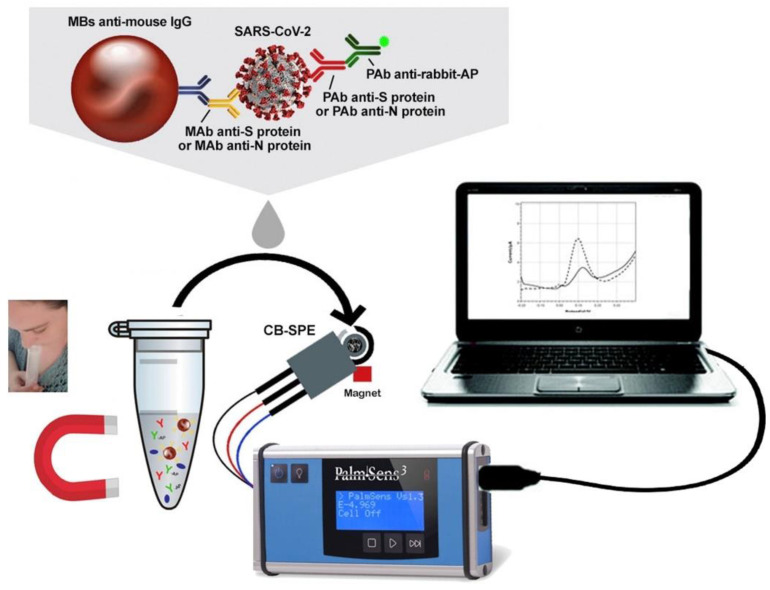
The MBs-based assay for SARS-CoV-2 detection in untreated saliva. Reprinted with permission from Elsevier from Ref. [152].

Still in the field of immunosensors, Fabiani et al. [152] proposed a screen-printed electrode modified with CB to detect SARS-CoV-2 in untreated saliva (schematically represented in Figure 12). For that, they used magnetic beads as a support and obtained a reliable and miniaturized electrochemical immunosensor. The immunosensor was able to detect the spike (S) protein or nucleocapsid (N) protein. As a result, they developed a good biosensor in terms of sensitivity, accuracy, and selectivity with the time of analysis, in addition to ease of use.

**Table 3:** Electrochemical sensors based on carbon black SPEs.

Analyte	Electrode *	Technique *	Linear Range (µmol L^−1^)	LOD (µmol L^−1^)	Samples	Reference
Aflatoxin B1	CB-SPE	DPV	1.06 × 10^−4^–2.35 × 10^−3^ (buffer) and 1.82 × 10^−4^–4.99 × 10^−3^ (extract)	4.16 × 10^−5^ (buffer) and 7.68 × 10^−5^ (extract)	Corn extract	[145]
Domoic acid (DOA)	CB-SPE	Amperometry	1.61 × 10^−2^–1.99 × 10^−2^ (buffer) 1.61 × 10^−2^–1.86 × 10^−1^ (scallop extract)	1.28 × 10^−3^ (buffer) 2.25 × 10^−3^ (buffer)	Buffer and Scallop extract	[142]
Dopamine (DA) and Epinephrine (EP)	aGO/CB-OMNiDIP-adduct/SPCE	DPV	7.57 × 10^−4^–4.07 × 10^−2^ (DA) and 4.04 × 10^−4^–9.99 × 10^−3^ (EP)	1.83 × 10^−4^–3.98 × 10^−4^ (DA) 9.28 × 10^−5^–1.09 × 10^−4^ (EP)	Aqueous, blood serum, urine and pharmaceutical	[151]
Dopamine (DA) Epinephrine (EP) and acetaminophen (ACP)	SPCE/CB-ERGO	SWV	4.9–19 (DA) 9.9–95 (EP) 9.9–95 (ACP)	4.1 × 10^−1^ (DA) 1.8 (EP) 1.5 (ACP)	Buffer	[150]
Levofloxacin (LVF) and acetaminophen (ACP)	CB(BP4750)-SPE	SWV	0.90–70.0 (LVF) 4.0–80.0 (ACP)	0.42 (LVF) 2.6 (ACP)	River water	[147]
Na^+^ ions	CB-SPE	DPV	1.0 × 10^3^ and 1.0 × 10^6^	63	Sweat	[144]
O-diphenols hydroxyty rosol (OLEU) and oleuropei (HYT)	CB-MoS_2_-SPE	DPV	0.3–30 (OLEU) 2–100 (HYT)	0.1 (OLEU) 1 (HYT)	Olive oil	[148]
Okadaic acid (OA) and domoic acid (DOA)	CB-SPE	DPV	1.28 × 10^−2^–2.31 × 10^−1^ (DOA in buffer), 1.28 × 10^−2^–1.09 × 10^−1^ (DOA in mussel extract), 3.35 × 10^−4^–4.10 × 10^−3^ (OA in buffer) and 4.35 × 10^−4^–4.84 × 10^−3^ (OA in mussel extract)	5.46 × 10^−3^ (DOA in buffer), 6.10 × 10^−3^ (DOA in mussel extract), 1.86 × 10^−4^ (OA in buffer), and 2.24 × 10^−4^ (OA in mussel extract)	Buffer and Mussel Extract	[143]
Spike protein (S) and nucleocapsid protein (N)	CB-SPE	DPV	---	19 ng mL^−1^ (S) 8 ng mL^−1^ (N)	Saliva	[152]
Uric acid	CB-GO-SPCE	Flow injection amperometry	0.05–2000	0.01	Urine	[149]

* The explanations of the abbreviations in Table 3 can be found in the Supplementary Materials.

### 3.4. Carbon Quantum Dot SPEs

The carbon quantum dots (CQDs) show high efficiency because the quantitative confinement and edge effect give rise to electronic and photoelectronic properties, an enlarged surface to mass, and a high conductivity. The CQDs have zero dimension and are derived from the carbon family, with properties derived both from graphene (GQDs, graphene quantum dots) and also from other carbon points [153]. GQDs are honeycomb-shaped graphene nanosheets with zero dimensions and a very compact size (<100 nm) and are generally the most synthesized for use in SPE [154]. For its synthesis, two approaches are generally used, namely bottom-up and top-down. Bottom-up involves the carbonization of an organic precursor through heat treatment, which is more advantageous as it allows for the precise control of the size and also for easier operation; the GQDs obtained are generally more soluble in water, with greater purity and a lower cost. The top-down method consists of the nanometric sculpture of carbon materials through logs, physical or chemical [155]. In this way, GQDs are usually synthesized bottom-up via the direct pyrolysis of citric acid [154,156,157].

#### 3.4.1. Carcinoembryonic Antigen

GQD-based SPEs have been most often used as SPE-electrochemical sensors (Table 4), and their merits have been improved with the combined use with metals such as copper, gold, or platinum, as in the case of the work developed by Mazloum-Ardakani et al. [153], in which gold nanoparticles with GQD were used for the modification of aptasensors for the detection of carcinoembryonic antigen. The GQD with AuNPs provided an improvement in the electrode’s performance by increasing its surface area and also improving the electrical properties of the electrode, making it possible to detect different types of cancer.

#### 3.4.2. Food Additives

Durán and collaborators [158] presented the detection of vanillin in foods using an SPE modified with both AuNPs and GQDs. The used GQDs were synthesized by a bottom-up approach, specifically acid thermal treatment. The electrochemical method was optimized and applied to several food samples with good LODs and satisfactory recoveries. The work developed by Mehmandoust et al. [105] proposed an electrode to determine the azo dye tartrazine using an SPCE modified by the deposition of porous reduced graphene oxide decorated with CQDs and platinum nanoparticles. In this way, this composite was used to manufacture SPEs with synergistic effects to enhance their electrochemical performance. This modification was made by drop-casting its suspension on the surface of the electrode, and from that, it was possible to sensitively determine traces of tartrazine with a good LOD and two wide linear ranges. The method was applied to sweets, soft drinks, gelatin, and water samples.

#### 3.4.3. Glucose

Nashruddin et al. [153] conducted a study for glucose detection using an SPE modified with GQDs and titanium carbide (Ti_3_C_2_), in which they helped to improve electrochemical behavior and analytical responses, such as sensitivity and stability. Titanium carbide aligned with GQDs are great for the production of sensors due to their synergistic effect and low interfacial resistance, which improves the electrocatalytic activity for glucose detection.

#### 3.4.4. Ifosfamide

Despite the advantages of metallic QDs, depending on the metal used, they can bring problems to the technological proposal. The traditionally used quantum dots consisting of heavy metals such as cadmium increase the environmental risks due to their toxicity. In this context, the development of ecologically sustainable alternatives has accelerated advances in the creation of QDs from less toxic materials. In the work developed by Prasad, Kumar, and Singh [159], GQDs were used since, in addition to their excellent characteristics such as large surface area, fast electron transfer kinetics, and excellent electrical conductivity intrinsic to graphene, quantum dots overcome the problems of the hydrophobic nature of the material due to the carboxylic portions on the edges, increasing the absorption of the analyte on the electrode’s surface. For this work, still seeking to overcome problems such as film formation and improve solubility, a monomer (N-Acryloyl-4-amino benzamide) was used to stabilize the GQDs. Thus, the manufactured sensor was applied for ifosfamide detection in real samples, without any matrix effect, cross-reactivity, or false positives.

#### 3.4.5. Dopamine, Tyrosine, Theophylline, Ascorbic Acid, Uric Acid

The simultaneous determination of some compounds can be a great challenge for electrochemical sensors; analytes such as ascorbic acid, dopamine, and uric acid are species that are mostly concomitant in living systems and samples, requiring the use of selective methodologies for the investigation of these biomarkers. In this regard, the use of materials such as QDs aligned with other composites that improve the analytical responses of the SPEs, making them more sensitive and selective, is a current trend. Beitollahi and collaborators [154] developed one SPE modified with GQDs, obtained through direct pyrolysis of citric acid. This modification architecture was efficient for the simultaneous determination of dopamine and tyrosine. Ganjali et al. [160] also used only GQD modification for the determination and detection of theophylline. In this same context, the work by Kunpatee et. al. [161] developed one SPCE modified with GQDs and ionic liquid (IL), which is a compound of various organic cations and inorganic anions, exists in liquid form at room temperature, and gives the system properties such as high thermal stability, high ionic conductivity, wide electrochemical window, and biocompatibility, functionalizing the GQDs. Thus, it was possible to individually and simultaneously determine ascorbic acid, dopamine, and uric acid.

Seeking to improve the intrinsic properties of GQDs, Aoun’s work [155] carried out the microwave-assisted doping of GQDs with chitosan and nitrogen, since the addition of N would provide an improvement in electrocatalytic activities as it would act directly on the oxygen and H_2_O_2_ reduction reaction, and the addition of chitosan showed a significant impact on the avoidance of the interferences commonly reported with AA and UA in the detection of DA, which was successfully performed for this system.

#### 3.4.6. Other Species of Interest

Like the previous work, Punrat et al. [162] used a polymer to improve the properties of GQDs. Polyaniline was added by electropolymerized aniline monomer and mixing GQDs, which were bottom-up synthesized from citric acid, on carbon SPE for Cr(VI) detection.

Ayad et al. [163] developed a range of SPE sensors with lab-made conductive carbon ink on a recycled X-ray sheet. Carbon QDs were synthesized from dextrose as a carbon precursor. During the development of the systems, it could be verified that the sensors modified with the CQDs presented wider linear ranges and greater sensitivity, which allowed for the selective detection of gemifloxacin in the presence of possible interferences and pharmaceutical formulations, and also in environmental waters.

**Figure 13:**
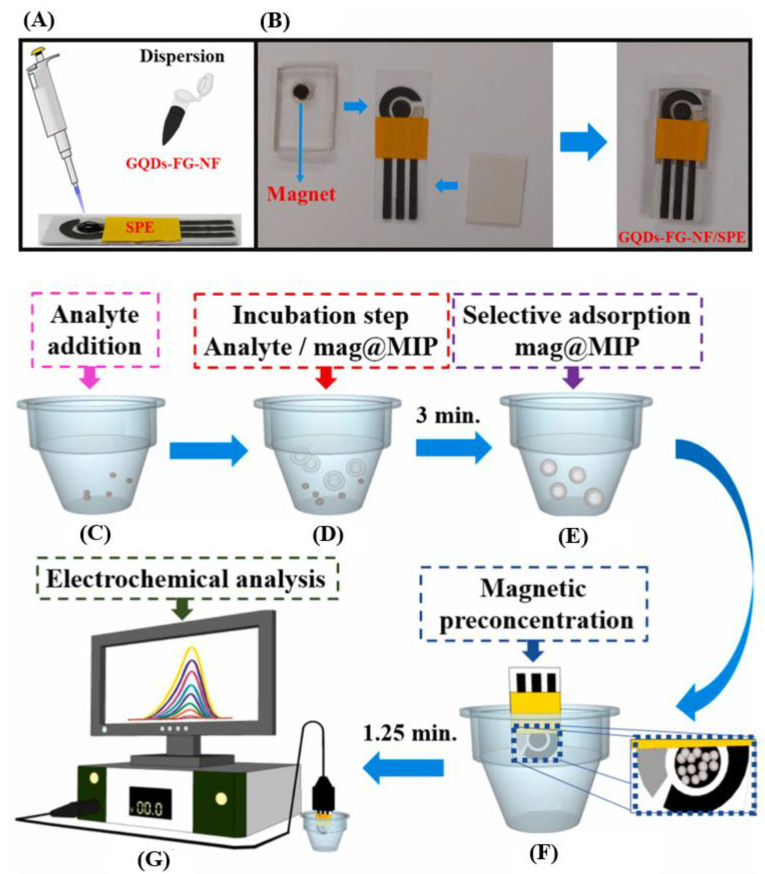
(**A**) SPE modification with GQDs-FG-NF and (**B**) fixing the magnet to the modified SPE. Sensor mechanism: (**C**) addition of the analyte; (**D**) incubation step with the mag@MIP in the analyte solution; (**E**) analyte adsorption by the mag@MIP; (**F**) separation of the analyte-mag@MIP with the electrode magnet (GQDs-FG-NF/SPE), and (**G**) steps of electrochemical analysis. Reprinted with permission from Elsevier from Ref. [156].

In addition to the use of high-cost metals, the work by Santos et al. [156] shows that magnetic nanoparticles can provide interesting features when combined with QDs. In this context, the work reports the development of an SPE modified with QD and magnetic nanoparticles (Fe_3_O_4_) coated with molecularly imprinted polymers for the selective detection of ethinyl estradiol in biological and environmental samples, as shown in Figure 13. In the same context, Canevari et al. [164] used magnetic nanoparticles hybridized with CQD for the determination of NADH in serum samples. The magnetization of these compounds, aligned with outstanding electrochemical properties for the modification of SPE, reports an interesting strategy for confining these compounds to the surface using a small magnet external to the system, improving its performance and sensitivity.

Finally, in addition to the optimal characteristics provided by CQDs for the creation of SPE sensory systems, these can also be used to enable the design of biosensors. Mollarasouli et al. [165] developed a label-free electrochemical immunosensor for the selective and sensitive determination of receptor tyrosine kinase in human serum. The disposable immunodetection platform was prepared by immobilizing the specific anti-AXL antibody on amine-functionalized GQDs in SPE sensors. Thus, the developed immunosensor was successfully applied in the serum of patients with heart failure.

**Table 4:** Electrochemical sensors based on carbon quantum dots SPEs.

Analyte	Electrode	Technique	Linear Range (µmol L^−1^)	LOD (µmol L^−1^)	Samples	Reference
AFP	CdS QDs	ASV differential pulse	5–500	4.9	Human blood serum	[166]
Antimicrobial resistance (RAM) GEMI	1 to 7	DPV	1, 3 and 4: 10–1.0 × 10^4^; 2: 1–1.0 × 10^3^; 5, 6 and 7: 1–1.0 ×10^4^	0.21	Pharmaceutical formulation and water	[163]
Ascorbic acid, dopamine, and uric acid	GQDs/IL–SPCE	DPV	25–400; 0.2–10 and 0.5–20	6.64; 0.06 and 0.03	Vitamin C tablets, dopamine injection	[161]
Carcinoembryonic (CEA)	Bio AuNP/Pol/Cu_2_O–CD/SPE	DPV, EIS, CV and Chronoamperometry	3.67 × 10^6^–3.67 × 10^3^	0.697	Human blood serum and pharmaceutical formulations	[167]
CEA	CdS QDs	ASV differential pulse	5–500	3.0	Human blood serum	[166]
Chromium	PANI/GQD/SPCE	SWV	0.05–5	0.005	Water	[162]
Clozapine	Go/Fe_3_O_4_/SiO_2_ nanocomposite	DPV	0.10–700	0.03	Urine and clozapine tablet	[168]
Diethylstilbestrol (DES)	GQD/SPE	LSV	0.05–7.5	8.8 × 10^−3^	Synthetic urine, tap water	[169]
Dopamine	CS/N, GQDs@SPCE	CV DPV	1–100 and 100–200	0.145	Human urine	[155]
Dopamine and tyrosine	GQD/SPE	CV DPV	0.1–1000 and 1.0–900	0.05 and 0.5	Human urine	[154]
Ethinylestradiol	(mag@MIP)–GQDs–FG–NF/SPE	CV SWV	1.0 × 10^−2^–2.5	2.6 × 10^−3^	Water, serum, and urine	[156]
Glycose	PEDOT:PSS/Ti_3_C_2_/GQD	DPV	0–500	65	Human blood serum	[153]
ICG	CdS QDs	ASV differential pulse	1–1.0 × 10^−2^	0.9	Human blood serum	[166]
Ifosfamide	m–GQDs–MIP	DPASV	---	4.2 × 10^−4^	Blood plasma, urine, and pharmaceutical formulations	[159]
NaDH	MagNP/C–dots/SPE	DPV	0.2–5	0.15	Serum	[164]
Progesterone	GQDs–NiO–AuNFs/f–MWCNTs (SPCE)	CV DPV	1.0 × 10^−4^–1	1.86	Human blood serum and pharmaceutical formulations	[170]
Solatol	MIP/AuNPs/GQD–SH/SPCE	DPV	0.1–250	0.035	Blood serum and tablets	[171]
Tartrazine dye (TRT)	Pt/CQDs@rGO/SPCE	DPV	0.01–1.57 and 1.57–9.3	7.93 × 10^−3^	Candy, soft drinks, jelly powder, and water	[105]
Theophylline	GQD/SPEs	CV DPV Chronoamperometry	1–700	0.2	Theophylline oral solution and urine	[160]
Tyrosine kinase	GQDs/SPCE	DPV	---	1.84 × 10^6^	Human blood serum	[165]
Vanillin	GQD@Nafion/AuNP–SPCE	LSV DPV	0.66–33	3.2	White-milk chocolate, custards, and sugar	[158]

The explanations of the abbreviations in Table 4 can be found in the Supplementary Materials.

### 3.5. Other Materials

In addition to the materials already described, other carbon materials with equivalent and/or superior characteristics have been used in the application of electrochemical sensing based on SPEs in different fields of analysis.

#### 3.5.1. SPE Modified with Graphitic Carbon Nitride

Graphitic carbon nitride (GCN) is a binary compound that is two-dimensional and stable under ambient conditions. This material has been applied to the development of electrochemical (bio)sensors due to its strong character as an electron donor of nitrogen and also because it provides more active reaction sites, which amplifies the electrochemical response [172]. Due to these characteristics, Jahani et al. [173] proposed the development of a sensitive and selective SPE modified with carbon nitride and graphite. Of simple construction, the sensor made possible the determination of amaranth dye within a good linear response range and low sensitivity. The GCN significantly increased the catalytic activity of the modified SPE; moreover, it provided good recoveries when applied to real samples, affirming the applicability of the developed sensor [173]. Nataraj et al. [174] also used graphitic carbon nitride in the modification of an SPCE for the detection of the pesticide carbendazim. In addition, the electrode contained selenium in its modification. The selenium GCN was prepared by a simple thermal polymerization method that allowed for the high sensitivity and efficiency of the formed SPCE, showing it to be an alternative in the analysis of pesticides harmful to health and the environment [174].

#### 3.5.2. SPE Modified with Nanospheres

In the last decades, the interest in the use of nanospheres in electrochemistry has intensified. These nanomaterials exhibit a large active surface area and high electrical conductivity. Therefore, Li et al. [175] developed a screen-printed gold electrode aptasensor modified by quantum dot-coated silica nanospheres for the detection of thrombin. The higher stability of the aptamers, coupled with the high sensitivity and high surface area of the nanostructures, allowed for optimal results for the proposed aptasensor [175].

#### 3.5.3. SPE Modified with Biochar

**Figure 14:**
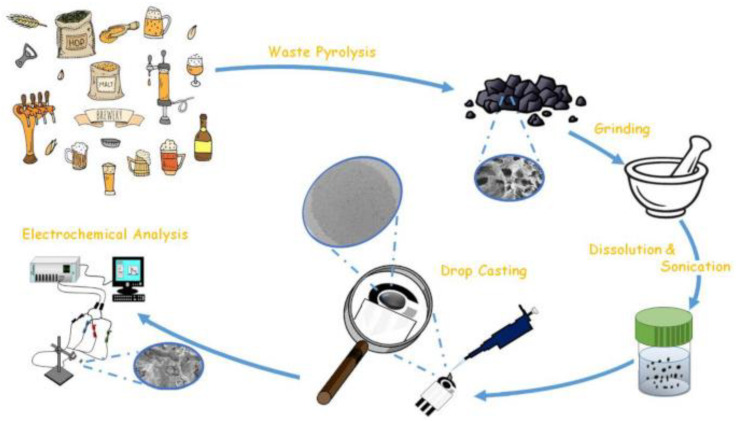
Biochar/SPE preparation scheme. Reprinted with the permission of Elsevier from Ref. [176].

Considering that the new research developed follows the ideas of green chemistry, with the reduction and/or reuse of generated waste, the use of carbon nanomaterials derived from biomass has drawn attention to the development of electrochemical sensors. In addition to all the good characteristics already presented for carbon nanomaterials, those derived from biomass still have the advantage of coming from cheap precursors, are of great abundance, have fast regeneration, have easy access, and are environmentally friendly. Because of this, brewery waste biochar was studied as an SPE modifier material [176]. Pyrolytic microgassing of the brewery waste pellets was performed, and after the manual grinding of the biochar samples, the material was ready for modification into SPEs. An overview of the work can be seen in Figure 14. The electrochemical responses obtained with the biochar/SPE showed a comparable performance to those of an SPE modified with commercial graphene, showing the possibility of the application of biochar as an environmentally friendly alternative to graphene.

In view of the works cited, it is observed that more carbonaceous materials have been studied and evaluated as modifiers of SPEs. These new studies point to new possibilities for SPEs, which can work with low-cost materials, employing green and circular economy principles.

### 3.6. Comparisons between Carbonaceous Nanomaterials

The carbon nanomaterials mentioned above are widely used in the fabrication of SPEs because they generally allow an increase in the electron transfer rates, which increases the sensitivity of the SPE analysis. Another characteristic of these nanomaterials is their ability to enable the construction of small (bio)sensors. In addition, the high electron transfer rate provided by these nanomaterials reduces fouling problems on the electrode surface [177].

The electronic properties of CNTs are strongly influenced by the curvature and diameter of the graphene sheets. They exhibit mechanical stability, withstanding current densities of up to 10^10^ A cm^−2^, which is ~3–4 orders of magnitude higher than that of metals [178]. One characteristic that differentiates nanotubes from other carbonaceous materials is the presence of a hollow cavity, which can serve as a growth site for different types of materials (metals, oxides, fullerenes, polymers, etc.) [179]. One limitation of working with CNTs is the presence of impurities on their surface, which can alter their properties. Therefore, treatment with an acid or alkaline solution is necessary to remove the impurities [180]. Regarding graphene, one of the main bottlenecks for its use is the development of methods and the large-scale production of samples with structural quality and control of the number of layers. Another current challenge is related to the toxicity of CNTs and graphene. The possible toxicity of these materials is correlated with their structure, chirality, length, surface area, possible metal contamination with catalysts, and the functionalization of their surface or lack thereof [178]. CQDs exhibit high chemical stability, environmental compatibility, low toxicity, and ease of large-scale synthesis at low cost, comparable to quantum dots. The electrochemistry of CQDs can be compared to that of GO sheets, because in the existence of oxygenated functional groups, these groups become responsible for the disorder of the sp^2^ carbon conducting network in the basal plane, resulting in impaired electron transfer [181]. CB finds applications as a filler and as a sensitive material, and it is economical [182].

Cinti et al. [182], conducted a study evaluating the physical and electrochemical properties of SPEs modified by different carbon nanomaterials. Characterizations by scanning electron microscopy (SEM) revealed that in CB-SPE, the CBs completely and uniformly reversed the electrode surface. In SWCNTs-SPE, the SWCNTs distributed themselves randomly and formed spongy clusters on the SPE. In GO-SPE, on the other hand, the formation of a continuous and smooth GO film on the SPE was observed. In the electrochemical analysis performed with [Fe(CN)6]^4−/3−^, at 0.05 V s^−1^, the CB-SPE, SWCNTs-O-SPE, rGO-SPE, and GO-SPE electrodes showed ΔE equal to 80, 100, 130 and 120 mV, respectively. CB-SPE showed an improvement in terms of overpotential reduction. The CB dispersion was also characterized as having higher stability compared to other carbonaceous dispersions. The CB dispersion was stable for at least two weeks, whereas the SWCNTs and GO dispersions no longer showed homogeneity the day after production.

Thus, it is observed that, in general, the nanomaterials are stable, which allows for their manipulation for the development of SPEs, but attention should be given to the possible toxicity of the materials to be used. Regarding the type of carbonaceous material to be used, it is worth evaluating their availability and cost, and which among the options can give a better design for the proposed SPE.

## 4. Conclusions and Future Perspectives

As one of the main innovations in modern electroanalysis, SPEs continue to be strong candidates in the expansion of electrochemical methods. In several of the works reviewed, characteristics that are always striking and highlighted are the reduction in the dimensions of the electrochemical analysis systems, portability, and disposability, allowing for the use of a few microliters of sample to perform an analysis of interest, whether to quantify biomarkers in human body fluids or even to monitor the contamination of water resources by metals or pesticides. This is in line with the principles of Green Analytical Chemistry [183] and the recently published 10 principles of Green Sample Preparation [184], which include in situ sample preparation, reducing the use of toxic solvents, preventing or minimizing waste generation, smaller sample sizes, enhanced sample throughput, etc. Looking towards the use of carbon nanomaterials in the preparation of SPEs, the most diverse nanostructures have been employed, which were separately discussed: graphenes, carbon nanotubes, carbon black, carbon quantum dots, and others (especially graphitic carbon nitride and biochar). These nanostructures are generally incorporated into the working electrode of the SPE design, using methods such as the classic drop-casting of material dispersion, or even magnetic attraction when subjected to functionalization with magnetic nanoparticles. In the latter case, there are several reports combining one or more carbon nanomaterials with metallic nanoparticles, ionic liquids, conductive polymers, biological agents, etc. All proposed modifications aim to ensure, in addition to the already emphasized typical characteristics of any SPE, high analytical performance (high analytical sensitivity, wide linear range, and low limit of detection and limit of quantification). The fields of application are varied, with contributions to the areas of environmental control, pharmaceuticals, biological analysis, forensics, and food quality. Although well explored, studies on SPEs based on carbon nanomaterials are far from being considered saturated. In view of this research, there is still room for proposing new modifications combining green routes for the synthesis of nanomaterials, more sustainable solvents for the preparation of conductive inks (for example, deep eutectic solvents, a hot topic in analytical chemistry), alternative substrates (recyclable materials), and carbon nanomaterials from biomass residues (contributing to reducing manufacturing costs and adding value to agro-industry waste). In addition, SPEs are outstanding options for configuring electrochemical detection systems in microfluidic devices and 3D-printed devices, another current trend in analytical chemistry. Some technical issues should also be pursued in future work, such as ensuring the repeatability in the mass production of SPEs, maintenance of the electrochemical response under (bio)fouling conditions when dealing with complex samples, and more complete analytical validations.

## Data Availability

Not applicable.

## Supplementary Materials

The following supporting information can be downloaded at: https://www.mdpi.com/article/10.3390/bios13040453/s1, the abbreviations.
