# Supplementary material for: Carbon Nanomaterials-Based Screen-Printed Electrodes for Sensing Applications
Source: Biosensors (Basel). 2023 Apr 3;13(4):453. doi: 10.3390/bios13040453 (PMC10136782; doi:10.3390/bios13040453)
Supplement: Supplementary file 1 [file biosensors-13-00453-s001.zip › biosensors-2297262-supplementary.pdf]

biosensors

Supplementary Material

Carbon Nanomaterials-Based Screen-Printed Electrodes for Sensing Applications

Rafael Matias Silva [1], Alexsandra Dias da Silva [1], Jéssica Rocha Camargo [2], Bruna Santos de Castro [1], Laís Muniz Meireles [3], Patrícia Soares Silva [1], Bruno Campos Janegitz [2] and Tiago Almeida Silva [1,*]

Explanations of the abbreviations in Table 1:

[Ni(dmgH$_2$]: Nickel (II)-Dimethylglyoxime complexe;

2D-g-C3N4/SDS/GNPs/SPE: 2-dimensional graphitic carbon nitride/sodium dodecyl sulfate/graphene nanoplatelets on the screen-printed electrodes;

AdTDPV-ERGO-SPEs: adsorptive transfer stripping differential pulse voltammetry in graphene oxide was electrochemically reduced on SPEs;

Ag/AgVO$_3$/N-rGO/SPCE: silver vanadate nanorods with silver nanoparticles decorated on the surface of the rods were synthesized by using a simple hydrothermal technique and later anchored onto nitrogen-doped reduced graphene oxide on the screen-printed electrodes;

AgNPs/GO/SPE: silver nanoparticles and graphene oxide modified on a screen-printed silver electrode;

Ag/rGO/ β-CD/SPCE: silver nanoparticles and reduced graphene oxide modified by β-Cyclodextrin on the screen-printed carbon electrode;

Ag-TiO$_2$-rGO/SPE: silver nanoparticles and titanium oxide and reduced graphene oxide modified screen-printed electrode;

AIR-GOC/SPE: atrazine imprinted reduced graphene oxide composite to modify the surface of a screen-printed electrode;

ASV: Anodic stripping voltammetry;

Au/L-cys/Fe$_3$O$_4$/RGO: gold nanoparticles and L-cysteine and iron(II, III) oxide on the screen-printed carbon electrode;

Au/NiO/rGO/SPCE: gold nanoparticles, nickel(II) oxide nanoparticles, and reduced graphene oxide on the screen-printed carbon electrode;

AuNP/PEI/RGO/SPE: gold nanoparticle/polyethyleneimine/reduced graphene oxide modified disposable screen-printed electrode;

AuNPs/AMBI/rGO/SPCE: gold nanoparticles, 5-Amino-2-mercaptobenzimidazole, and reduced graphene oxide on screen-printing carbon electrode;

AuNP-VS$_2$-rGO/SPCEs: gold nanoparticles decorating vanadium sulfide-reduced graphene oxide sheets on screen-printed carbon electrode;

Bi/LC-rGO/DSPE: bismuth film, L-cysteine, and reduced graphene oxide on the disposable screen-printed electrode;

(BiO)2CO$_3$-rGO-Nafion: bismuthite, reduced graphene oxide, and Nafion on screen-printed electrodes;

BTA-rGO/SPCE: organic heterocyclic benzotriazole was incorporated onto the reduced graphene oxide on a screen-printed carbon electrode;

C$_{60}$-rGO-NF/SPE: fullerene, reduced graphene oxide, and Nafion on the screen-printed electrodes;

CA: chronoamperometry;

CdTe-GO/SPE: cadmium telluride quantum dots and graphene oxide composite materials on screen-printed electrodes;

ChOx/Pt/rGO/P$_3$ABA/SPCE: cholesterol oxidase/platinum/reduced graphene oxide/poly(3-aminobenzoic acid) (Pt/rGO/P$_3$ABA) nanocomposite film on a screen-printed carbon electrode;

Co@MoS$_2$/rGO/SPE: cobalt oxides functionalized molybdenum disulfide/reduced graphene oxide on the screen-printed electrode;

CoMn2O$_4$RGO/SPCE: mixed oxide spinel of cobalt and manganese embedded reduced graphene oxide nanocomposite over the surface of modified screen-printed carbon electrode;

CoOOH-rGO/SPCE: cobalt oxyhydroxide nanoflakes and reduced graphene oxide over the surface of the screen-printed carbon electrode;

CuII-GO/SPCE: copper (II) ions-graphene oxide nanocomplex-modified screen-printed carbon electrode;

CuBi2O4/rGO@MoS$_2$/SPCE: copper bismuth oxide, molybdenum disulfide nanocomposite and reduced graphene oxide printed on screen-printed carbon electrode;

CuO/GO/SPCE: copper oxide and graphene oxide on screen-printed carbon electrode;

CV: cyclic voltammetry;

DPV: differential pulse voltammetry;

EIS: electrochemical impedance spectroscopy;

ERGO/β-CD/CdS/SPCE: reduced graphene oxide/β-cyclodextrin/cadmium sulfide composite on the screen-printed carbon electrode;

ERGO-AuNPCCAgPPE: electrochemically reduced graphene oxide with gold nanoparticle and printing of Ag–NP ink and the prepared printed paper-based electrode;

ErGO + AuNUs: nanocomposite of graphene oxide and gold nanourchins modify the surface of a screen-printed carbon electrode;

ERGO/MWCNTs/SPCEs: reduced graphene oxide/multiwalled carbon nanotubes/modified screen-printed carbon electrodes;

ERGO-SPE: reduced graphene oxide modifies the surface of a screen-printed carbon electrode;

Er-GO-SPE/AuNPs: reduced graphene oxide and gold nanoparticles were used to modify the working surface of a screen-printed electrode;

Fe$_3$O$_4$/GO-MSPE: iron oxide magnetic nanoparticles doping on reduced graphene oxide to modify a magnetic screen-printed electrode;

g-C$_3$N$_4$/rGO/SPE: graphite-like carbon nitride/reduced graphene oxide composite was deposited on a screen-printed carbon electrode;

GO-AgNPs/SPCE: graphene oxide-silver nanoparticle composite deposited on a screen-printed carbon electrode;

GO/AuNPs/MPBA: reduced graphene oxide/gold nanoparticles/4-mercaptophenylboronic acid-composites deposited on screen-printed electrodes; **GO-CMF/SPCE:** cellulose microfiber modified graphene oxide deposited on the surface of a screen-printed carbon electrode;

GO/Fe$_3$O$_4$/MB/Ab$_2$/Ppy: graphene ox-ide/nano-iron oxide/methylene blue/secondary antibody/polypyrrole nanohybrids modified a screen-printed gold electrode;

GOx/AuNP/PANI/rGO/NH$_2$-MWCNTs: glucose oxidase/gold nanoparticles/polyaniline/reduced graphene oxide/amine terminated multiwall carbon nanotubes modified screen-printed carbon electrode;

GPC/GPC3apt/RGO-Hemin/AuNPs/SPE: Glypican-3/Glypican-3 ap-tamer/reduced graphene oxide-hemin nanocomposites/gold nanoparticles modified on the screen-printed electrode surface;

Graphene/rGOSPE/Pyr-NHS: graphene/1-Pyrenebutyric acid N-hydroxysuccinimide ester/reduced graphene oxide dual-layer screen-printed electrode;

LSV: linear sweep voltammetry;

M-GRNs/SPCE: pure graphene nanosheets on screen-printed carbon electrode;

MnFe$_2$O$_4$/RGO/SPCE: manganese ferrite/reduced graphene oxide composite modified on the surface of home-made screen-printed electrodes;

Mn$_2$O$_3$-rGO/SPCE: manganese oxide nanoparticle-decorated reduced graphene oxide modified screen-printed carbon electrodes;

NH_2-rGO/TEMPO-nanocellulose/SPE: amine-functionalized reduced graphene oxide/2,2,6,6-tetramethylpiperidin-1-yl)oxyl nanocrystalline cellulose modified screen-printed electrode;

$NiCo_2O_4$/RGO/SPE: mixed nickel-cobalt oxide and reduced graphene oxide on the screen-printed electrode;

PANINS@rGO/SPCE: interlayered Polyaniline nanosheets @reduced graphene oxide composite coated on screen-printed carbon electrode;

PDbS-rGO/SPCE: block copolymer, poly(DMAEMA-b-styrene) and reduced graphene oxide modifying a screen-printed carbon electrode; PEC: photoelectrochemical sensor;

P-rGO/SPCE: piperazine functionalized reduced graphene oxide on screen-printed carbon electrode;

Pt/CODs@rGO/SPCE: three-dimensional porous reduced graphene oxide decorated with carbon quantum dots and platinum nano-particles modifying a screen-printed carbon electrode;

PtNFs/rGO: platinum nanoflowers and reduced graphene oxide nanocomposite on the screen-printed carbon electrode surface; rGO-500/SPCE and of rGO-600/SPCE: reduced graphene oxide by using thermal annealing at 500 °C modifying a screen-printed carbon electrode and reduced graphene oxide by using thermal annealing at 600 °C modifying a screen-printed carbon electrode;

RGO/AgNWs/AgNPs/SPCE: reduced graphene oxide with the insertion of silver nanowires followed by the anchoring of silver nanoparticles on screen-printed carbon electrode;

rGO/Au/Apt/BSA/Mxene/cDNA-MB: reduced graphene oxide/gold nanoparticles/aptamer/bovine serum albumin/Mxene/complementary DNA labeled with methylene blue on a screen-printed carbon electrode;

rGO-AuNUs/SPCE: aptasensor with reduced graphene oxide and gold nano-urchins modifying a screen-printed carbon electrode;

rGO-Au-SPE: reduced graphene oxide and a thick layer of gold were used to modify the surface of the screen-printed three electrodes;

RGO/Au/SPE: reduced graphene oxide/gold composite modified screen-printed carbon electrode;

rGO-CeO_2@Cyt c hydrogel/SPE: reduced graphene oxide–cerium oxide nanocomposite and cytochrome c hydrogel-modified screen-printed electrodes;

RGO-Cu_2O/Fe_2O_3/SPGE: reduced graphene oxide modified with copper oxide and iron(III) oxide nanocomposite on the screen-printed graphite electrode;

rGO/DWCNTs/Oct-Fe_3O_4/Cs/SPAuE: reduced graphene oxide/double-walled carbon nanotubes/octahedral-Fe_3O_4/chitosan composite material modified screen-printed gold electrodes;

rGO-methionine/SPCE: reduced graphene oxide and methionine film-modified screen-printed carbon electrode;

rGO/MFO-2/SPCE: magnesium ferrites anchored on reduced graphene oxide forming hybrid nanocomposite modifying a screen-printed carbon paste electrode;

rGO/MWCNTs/Thi/AuNP: reduced graphene oxide, multiwalled carbon nanotubes, thionine, and gold nanoparticles on a screen-printed electrode;

rGO/NiBTC/SPCEs: reduced graphene oxide and metal-organic frameworks (MOFs) of NiBTC to modify screen-printed carbon electrodes;

rGO-PEDOT:PSS/SPCE: reduced graphene oxide interspersed with poly(3,4-ethylenedioxythiophene):poly(styrenesulfonate) used as a transducer material deposited on a screen-printed carbon electrode;

rGO-PPy-AuNPs/SPCE: nanocomposite of reduced graphene oxide, polypyrrole, and gold nanoparticles was immobilized on the screen-printed carbon electrode;

rGO-PPy-(L-Cys)-AuNPs/SPCE: nanocomposite of reduced graphene oxide, polypyrrole, and gold nanoparticles deposited on the surface of screen-printed carbon electrode;

rGO/PPy NTs-GSPE: nanocomposite of reduced graphene oxide/polypyrrole nanotubes to modify graphite screen-printed electrode;

rGO/SPCE: reduced graphene oxide-Nafion nanocomposite by modifying the surface of screen-printed carbon electrode;

SPCE-Amino-rGO/IL/Amino-MSNs: screen-printed carbon electrode was modified with a nanocomposite made from amino-reduced graphene oxide synthesized from natural graphite powder, an ionic liquid and amino-mesosilica nanoparticles;

SPCE-PANI-AuNPs: immune gold@platinum nanoparticles, neutral red, reduced graphene oxide nanocomposite and regenerative leucoemeraldine base polyaniline/gold nanoparticles modified screen-printed carbon electrode;

SPCE/rGO/PVA/anti-5mC/BSA/DNA-probe-Fe_3O_4-CA nanoparticles: reduced graphene oxide/polyvinylalcohol/antibody against 5-methylcytosine/bovine serum albumin/ssDNA probe conjugated to Fe_3O_4-citric acid nanocomposites on the modified screen-printed carbon electrode;

SPCEs/GO/cDNA-aptamer/3D-rGO-AuNPs: graphene oxide complementary DNA-linked-aptamer and hybridization of ap-tamer-linked 3D-reduced graphene oxide/gold nanoparticles on the modified screen-printed carbon electrode;

SPC-RGO: reduced graphene oxide on the screen-printed carbon electrode;

SPE(a)/rGO-AuNPs: molecularly imprinted poly(phenol) film modified with reduced graphene oxide and gold nanoparticles on the screen-printed electrode;

SPE/rGO/AuNPs: reduced graphene oxide/gold nanoparticles modified screen-printed electrode;

SPE/rGO-MWCNT, Au nanoparticle, L-cysteine: nanocomposite of reduced graphene oxide and multiwalled carbon nanotubes, gold nanoparticles, L-cysteine were electrodeposited on a screen-printed carbon electrode;

SPE/rGO-NHS-AuNFs: graphene oxide sheets crosslinked with N-hydroxysuccinimide and functionalized with gold nanoflowers modified a screen-printed electrode;

Sr-ZnO@rGO/SPCE: strontium doped zinc oxide with reduced graphene oxide modified a screen-printed carbon electrode;

SW-AdCSV: square-wave adsorptive cathodic stripping voltammetry;

SW-CSV: square wave cathodic stripping voltammetry;

SWV: square wave voltammetry;

TITU-rGO/CSPE: tris-thiourea and reduced graphene oxide sheets by modifying a miniaturized carbon paste screen-printed electrode;

WO_3/SPE: tungsten (VI) oxide rods modified screen-printed electrode;

$ZnFe_2O_4$/RGO/SPE: iron zinc oxide and reduced graphene oxide modified screen-printed electrode;

ZnO-NPs/rGO/SPCE: zinc oxide nanoparticles and reduced graphene oxide by modified screen-printed carbon electrode.

Explanations of the abbreviations in Table 2.

AdSWV: Adsorptive stripping wave voltammetry;

ASV: Anodic stripping voltammetry;

AuNPs/CNT/SPCE: gold nanoparticles decorated on carbon nanotube-modified screen-printed electrode;

CuF/GCE: copper film-modified glassy carbon electrode;

CuF/CN/SPE: copper film with carbon nanotubes modified screen-printed electrode;

DPAdSV: differential-pulse adsorptive stripping voltammetry;

DPASV: differential pulse anodic stripping voltammetry;

IL-SWCNT/SPCE: screen-printed single-walled carbon nanotubes with ionic liquid 1-butyl-3-metilimidazolium tetrafluorborate;

mMWCNTs/SPE: screen-printed electrode with magnetic nanoparticles and **MWCNTs; MWCNTs-COOH/SPCE**: screen-printed carbon electrodes modified with carboxy-functionalized multi-walled carbon nanotubes;

MWCNTs-CS/SPCE: screen-printed carbon electrode with MWCNTs and chitosan;

MWCNT/GP/SPCE: screen-printed carbon paste electrode combined with multi-walled carbon nanotubes and graphene;

OD600: optical density measured at a wavelength of 600 nm;

RGO-MWCNT-AuNP/SPE: Graphene oxide/multi-walled carbon nanotubes/gold nanoparticle hybrid functionalized disposable screen-printed carbon electrode;

SPCE: single-walled carbon nanotubes-modified screen-printed carbon electrode;

SPCE-CNT/Nafion: screen-printed carbon electrode with CNTs and Nafion;

SPCE/MWCNTs/BiF: screen-printed carbon sensor modified with multiwalled carbon nanotubes and bismuth film;

SPCE/MWCNTs-COOH: screen-printed carbon electrode modified with carboxyl functionalized multiwalled carbon nanotubes;

SPCE/PAKB NPs/CNTs: screen-printed carbon electrodes modified with polymeric nanoparticle-carbon nanotube composites;

SPGPHE: graphene-modified screen-printed electrode;

SPCNFE: carbon nanofibers modified screen-printed electrode;

SWSV: Square-wave stripping voltammetry;

SPCNTE: multi-walled carbon nanotubes modified screen-printed electrode.

Explanations of the abbreviations in Table 3.

aGO/CB-OMNiDIP-adduct/SPCE: acryloylated-graphene oxide/carbon black composite polymer was developed on the screen-printed carbon electrode;

CB(BP4750)-SPE: carbon black materials (BLACK PEARLS 4750) was developed on the screen-printed carbon electrode;

CB-GO-SPCE: carbon black and graphene oxide modified screen-printed carbon electrode;

CB-MoS_2-SPE: carbon black and molybdenum disulfide modified screen-printed carbon electrode;

CB-SPE: carbon black nanomaterial modified screen-printed carbon electrode;

SPCE/CB-ERGO: carbon black and electrochemically reduced graphene oxide composite on screen-printed carbon electrodes.

Explanations of the abbreviations in Table 4.

1 to 7: SPEs with the working electrode printed using homemade graphite ink for sensors 1–5 or homemade graphite-CQDs ink for sensors 6 and 7;

Bio–AuNP/Pol/Cu_2O–CD/SPE: electrochemical aptasensor based on conducting polymers and nanomaterials (modified carbon quantum dots and biosynthesized gold nanoparticles);

CS/N, GQDs@SPCE: doped graphene quantum dots-chitosan nanocomposite-modified nanostructured screen-printed carbon electrode;

G–QD@Nafion/AuNP–SPCE: gold nanoparticle-modified screen-printed carbon electrode modified with graphene quantum dots in a Nafion matrix;

GQDs–NiO–AuNFs/f–MWCNTs (SPCE): label-free electrochemical aptasensor based on GQDs–NiO-AuNFs/f-MWCNTs modified screen-printed carbon electrode (SPCE);

GQDs/IL–SPCE: graphene quantum dots (GQDs) and ionic liquid (IL) modified screen-printed carbon electrode;

GQDs/SPCE: immunosensor for AXL using SPCEs modified with amino-group functionalized GQDs;

GQD/SPEs: SPE modified by graphene quantum dot; m–GQDs–MIP: IFO-selective m-GQDs-MIP-based nanocomposite synthesized directly on the surface of SPCE;

(mag@MIP)–GQDs–FG–NF/SPE: SPE modified with functionalized graphene, graphene quantum dots, and molecularly printed polymer-coated magnetic nanoparticles;

MagNP/C–dots/SPE: SPE modified with superparamagnetic MagNP (Fe_3O_4) nanoparticles and carbon quantum dots;

MIP/AuNPs/GQD–SH/SPCE: molecular printed sensor constructed by modifying the SPCE with graphene thiol quantum dots (GQD-SH) and gold nanoparticles (AuNPs);

PANI/GQD/SPCE: polyaniline-graphene quantum dot-modified SPCE; Go/Fe_3O_4/SiO_2 nanocomposite: SPE modified with Go/Fe_3O_4/SiO_2 nanocomposite;

PEDOT:PSS/Ti_3C_2/GQD: SPE modified with nanocomposite of poly(3,4-ethylenedioxythiophene):polystyrene sulfonate, titanium carbide and graphene quantum dot;

Pt/CQDs@rGO/SPCE: screen-printed carbon electrode (SPCE) modified by depositing three-dimensional porous reduced graphene oxide decorated with carbon quantum dots and platinum nanoparticles.
